# Economic burden of migraine in Latvia and Lithuania: direct and indirect costs

**DOI:** 10.1186/s12889-019-7461-2

**Published:** 2019-09-09

**Authors:** Ágnes Lublóy

**Affiliations:** 1 Stockholm School of Economics in Riga, Strēlnieku iela 4a, Rīga, LV-1010 Latvia; 2 Corvinus University of Budapest, Fővám tér 8, Budapest, 1093 Hungary

**Keywords:** Migraine, Direct cost, Indirect cost, Health care resource utilization, Productivity loss, Reduced workforce participation, Absenteeism, Presenteeism, Latvia, Lithuania

## Abstract

**Background:**

Migraine is a primary headache disorder which affects all aspects of life. The financial burden of migraine imposed on the society might be substantial. This study aims at estimating the economic cost of migraine in Latvia and Lithuania, including both direct and indirect costs. Direct costs encompass the costs of migraine-related health care resource utilization. Indirect costs are related to productivity loss, the potential or expected earnings lost due to migraine.

**Methods:**

Direct cost is assessed by using the prevalence method, a widely used cost-of-illness approach. The prevalence rate of migraine and the migraine-related health care resource utilization are proxied from the literature, whereas unit cost of medical services and procedures are retrieved from national databases and providers. For estimating the indirect cost of migraine, we follow the human capital approach. We quantify three components of indirect costs: reduced labour force participation, absence from work and reduced productivity while at work. The number of unemployed migraineurs, days missed from work and days lost due to impairment while at work are drawn from the literature. Unemployment rate and average income in Latvia and Lithuania are then inserted to assess indirect costs.

**Results:**

We find that the mean per-person total cost of migraine is €801 annually in Latvia, and €721 in Lithuania. In both countries around 30% of total cost is direct cost; cost related to a wide array of migraine-related medical services and interventions. The total cost of migraine is €112.26 million in Latvia, corresponding to 0.42% of Latvia’s GDP. The total cost of migraine is €149.62 million in Lithuania, corresponding to 0.35% of Lithuania’s GDP. In both countries two thirds of total cost is related to lost workdays due to absenteeism and presenteeism.

**Conclusions:**

The financial burden of migraine imposed on the society is substantial in Latvia and Lithuania. Improvements in care for patients with migraine, such as easier access to structured headache assessment services, wider availability of various procedures and preventive medications would significantly increase direct costs. Nevertheless, this cost increase might be far outweighed by lower migraine-related productivity loss, especially as the prevalence of migraine is the highest in the most productive years of life.

**Electronic supplementary material:**

The online version of this article (10.1186/s12889-019-7461-2) contains supplementary material, which is available to authorized users.

## Background

Migraine is a common disabling primary headache disorder. Migraineurs suffer from intense headaches, the pain is typically a moderate or severe throbbing or pulsing sensation. Other symptoms commonly associated with migraine include nausea, vomiting, and blurred vision. Patients frequently experience several additional neurologic, gastrointestinal, and autonomic symptoms, such as diarrhoea, abdominal cramps, sweating, and increased sensitivity to light and sound [1]. These symptoms are often severe enough and affect all aspects of life—work, daily routines, social and leisure activities. Many epidemiological studies document the high prevalence and the socio-economic and personal impacts of migraine. In the Global Burden of Disease Study 2010, it was ranked as the third most prevalent disorder in the world [2]. In Global Burden of Disease Study 2015 it was ranked as the third-highest cause of disability worldwide in both males and females under the age of 50 years [3].

The financial burden of migraine imposed on the society as a whole is substantial. Migraine sufferers use health care resources more often than individuals without migraine; they visit their general practitioners more frequently, they typically consult a neurologist about their headaches and several diagnostic tests are performed to rule out other causes of migraine symptoms. Costs associated with migraine-related health care resource utilization are labelled as direct costs. Indirect costs are related to productivity loss caused by reduced labour force participation, absence from work, and reduced productivity while at work. As migraine prevalence typically peaks between the ages of 25 and 55, during the most productive years of a person’s life, productivity loss is of particular importance [4]. Several recent studies report that direct costs are relatively low in comparison with indirect costs [5–8].

This study aims at estimating the economic cost of migraine in Latvia and Lithuania. To the best of our knowledge no study so far has assessed the economic cost of migraine in Latvia, while one study has examined the cost of migraine in Lithuania as part of the Eurolight project [5]. The Eurolight project was a collaborative data-collection exercise in ten European countries, supported by the European Agency for Health and Consumers. Eurolight collected data on headache disorders in a cross-sectional questionnaire-based survey; data collection took place between November 2008 and August 2009, roughly 10 years ago. Thus, no up-to-date information is available on the burden associated with migraine in Latvia and Lithuania, despite migraine being one of the major causes of disability across the globe [3].

In this study we assess both direct and indirect costs. Direct costs encompass the cost of a wide array of migraine-related medical services and procedures: consultations with doctors, hospitalizations, emergency room visits, diagnostic testing, and medications. In the absence of primary data, we perform a targeted literature review to proxy the migraine-related health care resource utilization. Unit prices of health care services commonly used by migraineurs is then retrieved for Latvia and Lithuania.

Indirect costs cover the productivity loss related to reduced workforce participation, absenteeism and presenteeism. The disability and decreased functional status associated with migraine can be severe; migraine imposes substantial burden on affected individuals. The decreased functional capacity might be reflected in the disability to think clearly, lack of focus, loss of concentration and motivation, lack of energy to complete a task before deadline, and the unavoidable need to stop and rest. Several studies report that migraine sufferers require bed rest to relieve their pain [9, 10]. The productivity losses associated with migraine are significant, the indirect cost of migraine is considered to be far more important than direct costs [5, 6, 8]. For example, Linde et al. [5] report for Europe that indirect costs accounted for 93% of total cost of migraine.

To estimate indirect cost for employed migraineurs, we consider that the entire annual gross income is lost; this income could have been earned had the individual lived without disabling migraine. To estimate indirect cost for employed migraineurs, loss incurred through absenteeism and presenteeism should be determined. Absenteeism is defined as being absent from work as a result of headache, while presenteeism is defined as lost productivity due to headache. We perform a targeted literature review to proxy the number of days missed from work and the number of days lost due to impairment while at work. The number of lost workdays is then multiplied by the average daily income earned in Latvia and Lithuania.

## Methods

### Research design

In this study, we follow the best practice cost-of-illness approach [11, 12]. In medical literature cost of illness studies are widely used to measure all the costs of a particular disease, encompassing direct, indirect, and intangible costs [11]. Two methods are widely used for costing illness; the incidence and prevalence approaches. The incidence-based approach estimates the lifetime cost of incidents diagnosed in a particular year. In contrast, the prevalence method employed in this research is more popular; it assesses the total cost of a disease in a given year. In the prevalence method, the total cost estimate is the product of mean per patient cost and the prevalence rate. In this study total cost is measured on the level of the society, regardless whether incurred by the government, the individual or third party. This comprehensive perspective allows us to take all costs into account, also the ones that could have been shifted to another party instead of saving it.

**Fig. 1:**
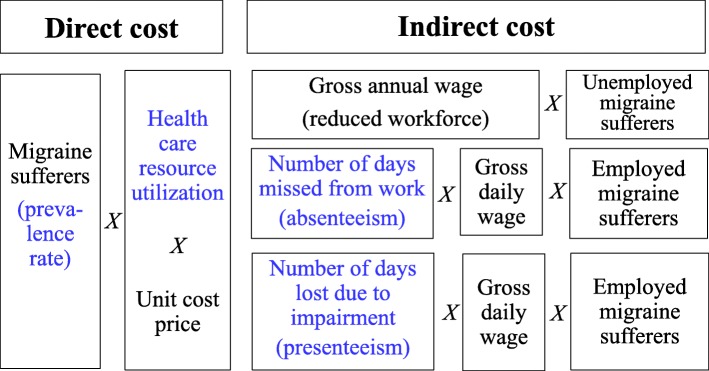
Research design of cost estimation. Items in blue are estimated from systematic or targeted literature reviews. Items in black are retrieved either from the statistical offices or from other, mostly public databases in Latvia and Lithuania

Figure 1 shows the research design employed in this study. For estimating the direct cost of migraine in Latvia and Lithuania, first migraine-related health care resource utilization is proxied by relying on previous literature. Per person direct cost is then derived as the product of the health care services used by migraineurs and the unit prices of those services. Health care resource utilization data captures both the proportion of migraine sufferers using a particular resource and the frequency of that resource use. Total direct cost is estimated by multiplying the per person direct cost with the number of patients suffering from migraine. The number of migraineurs is determined by using the prevalence rate, the proportion of people in a population who suffers from migraine in a particular period. We perform a systematic literature review to arrive at a reliable prevalence rate estimate for Latvia and Lithuania; meta-analysis estimates combining the findings of several European studies on migraine prevalence are preferred over single-country estimates.

For estimating the indirect cost of migraine in Latvia and Lithuania, we follow the human capital approach. This approach assesses productivity loss by calculating the potential or expected earnings lost due to migraine [13]. Three components contributing to productivity loss is quantified in this study. First, we estimate the loss related to reduced workforce participation; average annual gross income is considered as the per person loss incurred through unemployment. Although migraineurs are less likely to be employed than the general population [4, 14], we conservatively assume that the prevalence rate of migraine among employed and unemployed is the same. Second, among employed migraineurs we estimate the productivity loss incurred through absenteeism. The annual number of days missed from work is determined from literature review; this figure is then multiplied by the gross daily wage to arrive at a per person productivity loss due to absenteeism. Third, among employed migraineurs we calculate the productivity loss incurred through presenteeism. The annual number of days lost due to impairment is determined from the literature; this figure is then multiplied by the gross daily wage to arrive at a per person productivity loss due to presenteeism.

We report per person and total costs for health care resource utilization, reduced workforce participation, absenteeism and presenteeism. Total cost is reported both in million euros and as percentage of GDP. We deliver a conservative economic cost estimate; the prevalence rate of migraine and the number of lost workdays adopted from the literature can be considered as lower bounds for those items. Sensitivity tests are them performed with other, less conservative estimates.

### Data derived from the literature

#### Prevalence of migraine in Latvia and Lithuania

The economic cost of migraine in Latvia and Lithuania is estimated in line with the best practice cost-of-illness approach. This approach requires the estimation of the prevalence rate, the proportion of people in a population who suffer from migraine in the base period, in 2017.

The prevalence rate is estimated by identifying systematic reviews on migraine prevalence from a systematic review of the literature in PubMed and Scopus.^1^ From the 45 studies identified only three deemed relevant; these carefully executed, high-quality *systematic reviews* are listed in panel A of Table 1.^2^ For current prevalence, for Europe, Stovner et al. [15] estimated an arithmetic average migraine prevalence of 15% in their meta-analysis. For lifetime prevalence, for Europe, the authors reported an arithmetic average migraine prevalence of 17% [15]. In an updated study, Stovner and Andrée [17] estimated that the mean prevalence of current migraine is slightly lower, 14.7% in Europe. In contrast, Woldeamanuel and Cowan [18] derived a migraine prevalence rate of 11.4% for Europe. The difference in migraine prevalence between the two studies (14.7% in [17] vs 11.4% in [18]) can be explained by the facts that Woldeamanuel and Cowan [18] employed weighted averages and included more up-to-date studies; both factors render their estimates more reliable.

**Table 1:** Prevalence rate of migraine in various studies

Study	Methodology	Geographic coverage	Main findings	Migraine prevalence
Panel A: Systematic literature reviews				
Stovner et al. [15]	Population-based studies were included if they applied the International Headache Society criteria for migraine and tension-type headache [16]. In addition, studies on headache in general and on chronic daily headache met the inclusion criteria as well.	107 studies (Africa 8; Asia 20; Australia-Oceania 4; Europe 48; North-America 14; Central & South America 13)	Globally, the percentages of the adult population with an active headache disorder are 46% for headache in general, 11% for migraine, 42% for tension-type headache and 3% for chronic daily headache. For Europe, in case of migraine the authors report current prevalence rate of 15% (range 10 to 25%, estimate based on 14 studies) and lifetime prevalence rate of 17% (range 12 to 28%, estimate based on 10 studies).	11% (globally) 15% (Europe)
Stovner & Andrée [17]	Population-based studies of headache and migraine were included if they applied the International Classification of Headache Disorders (ICHD-1 or ICHD-2) for headache diagnosis. Only studies from Europe were reviewed.	33 studies (Europe)	The mean prevalence of current migraine among over 170,000 adults is 14.7% (8% in men and 17.6% in women). The lifetime prevalence rate is higher; 16% in adults (11% in man and 20% in women, respectively). If in addition to migraine without aura, migraine with aura, and probable migraine is also considered, the prevalence rate almost doubles.	14.7% (Europe)
Woldea-manuel & Cowan [18]	Community or population-based non-clinical studies on headache met the inclusion criteria in different countries worldwide.	302 studies (worldwide)	The global migraine prevalence rate among 6,216,995 participants is 11.6%. The migraine prevalence rate is 10.4% in Africa, 10.1% in Asia, 11.4% in Europe, 9.7% in North America and 16.4% in South America. The migraine prevalence is 13.8% in females, 6.9% in males. The prevalence rate of migraine is 11.2% in urban residents, 8.4% in rural residents.	11.6% (globally) 11.4% (Europe)
Panel B: Eurolight project				
Katsarava et al. [19]	Cross-sectional questionnaire-based surveys were employed. In six countries (Germany, Italy, Lithuania, Luxembourg, Netherlands, Spain), samples were population-based. In three countries (Austria, France, UK), general practitioners recruited consecutive patients visiting them for any reason. The sample in Ireland (and some additional samples in Spain and Netherlands) was recruited through lay organisations.	10 EU countries (Austria, France, Germany, Ireland, Italy, Lithuania, Luxembourg, Netherlands, Spain, United Kingdom)	Among 9247 participants (mean age 43.9 ± 13.9 years, male to female ratio 1:1.4), 3466 (37.6%) were diagnosed with migraine, either definite or probable. Of these, 1175 reported frequent migraine (> 5 days/month), representing 12.71% of the sample population. In population-based samples, minorities of participants with migraine had seen a general practitioner (9.5–18.0%) or specialist (3.1–15.0%), and smaller minorities received adequate treatment. Participants with migraine who had consulted specialists (3.1–33.8%) were receiving the best care; those treated by GPs (9.5–29.6%) fared less well, and those dependent on self-medication (48.0–84.2%) were, apparently, inadequately treated.	37.6% (Europe, definite or probable migraine) 12.71% (Europe, migraine on more than 5 days/month)
Panel C: Baltic countries				
Rastenytė et al. [20]	Cross sectional questionnaire-based surveys. The surveys were part of the Eurolight project using the same structured questionnaire in ten EU countries. The sample was population-based in Lithuania; adults in and around Kaunas were contacted. The sample reflected age (in range 18–65 years) and gender composition of Lithuania and proportions living in rural (33%) or urban (67%) areas. Participants were contacted by door-to-door cold-calling.	Lithuania	Based on 537 completed interviews, the gender-adjusted 1-year prevalence rates are 74.7% for any headache; 18.8% for migraine; 42.2% for tension-type headache; 8.6% for other headache on ≥15 days/month; and 3.2% for probable medication-overuse headache.	18.8% (Lithuania, definite or probable migraine)
Katsarava et al. [19]	Cross sectional questionnaire-based surveys. The surveys were part of the Eurolight project using the same structured questionnaire in ten EU countries. The sample was population-based in Lithuania; adults in and around Kaunas were contacted. The sample reflected age (in range 18–65 years) and gender composition of Lithuania and proportions living in rural (33%) or urban (67%) areas. Participants were contacted by door-to-door cold-calling.	Lithuania	From the 616 respondents 149 (24.19%) were diagnosed with migraine, either definite or probable. (The survey did not distinguish between patients meeting criteria for definite migraine or probable migraine.) In Lithuania, 62 participants reported frequent migraine (> 5 days/month), which corresponds to 10.06% (62/616) prevalence rate of frequent migraine. These rates are the observed ones, without gender adjustments.	24.19% (Lithuania, definite or probable migraine) 10.06% (Lithuania, migraine on more than 5 days/month)
Toom et al. [21]	Population-based random sample demographically representative of the Estonian population; respondents from Tartu city and Tartu county. The sample reflected gender, age (in range 18–65 years), marital status and educational level composition of Estonia, and proportions living in rural (37%) or urban (62%) areas. Telephone or face to face structured interviews.	Estonia	Among the 1215 respondents weighted one-year prevalence of migraine was 17.7%, either definite of probable. Weighted one-year prevalence of definite migraine, either episodic or chronic was 7.3%, while weighted one-year prevalence of probable migraine, either episodic or chronic was 10.4%.	17.7% all migraine; 16.8% episodic migraine (6.6% definite and 10.2% probable); 0.9% chronic migraine (0.7% definite and 0.2% probable)

In *Europe*, valuable prevalence data has been recently collected in the Eurolight project, an initiative supported by the European Commission Executive Agency for Health and Consumers [19]. Eurolight collected data on headache disorders in a cross-sectional questionnaire-based survey in ten European countries, which together represented over 60% of the adult population (18–65 years) of the European Union. The most important characteristics and the major findings of the Eurolight project is summarized in panel B of Table 1.

None of the systematic reviews shown in Panel A of Table 1 listed studies estimating prevalence rate of migraine in *the Baltic countries*. By undertaking a systematic search of the published literature in PubMed and Scopus, we concluded that no prevalence rate for migraine is available in Latvia, while three recent studies cover the other two Baltic countries.^3^ The Eurolight project assesses the prevalence rate of migraine in Lithuania [19, 20], while Toom et al. [21] report prevalence rate of migraine for Estonia. The most important characteristics and the major findings of studies covering the Baltic countries are summarized in Panel C of Table 1.

To conclude, it is reasonable to assume that the prevalence rate of migraine in the Baltics is close to those meta-analysis estimates which combine the findings of several European studies on migraine prevalence. The prevalence rate of 11.4% estimated by Woldeamanuel and Cowan [18] for Europe involving a sample size of 808,749 participants is used in further analysis as a conservative estimate. In sensitivity test the migraine prevalence rate of 12.71% from the Eurolight project for Europe is used as well [19]. The estimate from the Eurolight project is based on surveying 9247 individuals in ten EU countries and reflects migraine on more than 5 days/month.

For Lithuania, we have also considered using the gender-adjusted migraine prevalence rate of 18.8% from the Eurolight project [20]. This estimate is based on surveying 573 adults in Lithuania. Although the sample is population-based and has as reasonable sample size, the estimate classifies definite and probable migraine together as migraine; a drawback which let us exclude this estimate from further analysis. Similarly, we have also considered using the migraine prevalence rate of 17.7% reported for Estonia which is also a population-based estimate [21]. Although the authors distinguish between definite and probable migraine, the weighted prevalence rate of definite migraine is much smaller than in many other studies, only 7.3%, which again let us exclude this estimate from further analysis. In their study, the authors list several sources of possible underestimation [21].

#### Health care resource utilization

For estimating the direct cost of migraine in Latvia and Lithuania, first migraine-related health care resource utilization shall be estimated. A targeted literature review produced a number of valuable studies; in this subsection these studies are reviewed briefly with the aim of identifying the most relevant source to estimate the migraine-related health care resource utilization in Latvia and Lithuania.

Lantéri-Minet et al. [22] review systematically 34 studies of patients with chronic daily headache, occurring on at least 15 days per month. Although the authors focus on patients with chronic migraine to evaluate the evidence for quality of life impairment, disability, health care resource use and economic burden, they provide an excellent review of studies on resource utilization and economic impact covering several migraine types. The authors document that as of July 2009 resource utilization data was available only from two studies. The GRIM 2000 study is a large nationwide survey of headache characteristics and health care resource utilization in France [16], while the American Migraine Prevalence and Prevention (AMPP) study is a 5-year, longitudinal, national study of headache in the US [6]. Lantéri-Minet et al. [23] and Auray et al. [24] use data from the GRIM study, while Munakata et al. [6] builds upon the survey results of the AMPP study to access the total direct cost of migraine in France and in the US, respectively.

Lantéri-Minet et al. [22] review studies published before July 2009. Since that date, as identified by a targeted literature search, three large surveys have been carried out which can be considered as valuable source of information on migraine-related health care resource utilization: the first International Burden of Migraine Study (IBMS-I), the second International Burden of Migraine Study (IBMS-II), and the National Health and Wellness Survey. A detailed overview of the targeted, web-based methodology applied in IBMS-I is provided in [25]. In IBMS-I participants were recruited and surveyed in ten developed countries, the data collected covered sociodemographic and clinical characteristics, resource utilization, disability, health-related quality of life, anxiety, depression and productivity from over nine thousand participants. IBMS-II was also an international, web-based, cross-sectional survey; it investigated the burden of chronic and episodic migraine in six developed countries. IBMS-II targeted 600–600 patients with chronic and episodic migraine, respectively. In both IBMS-I and IBMS-II only participants meeting ICHD-2 criteria for migraine were included and they were classified as patients with chronic migraine (≥ 15 headache days per month) or episodic migraine (< 15 headache days per month) [25, 26]. The National Health and Wellness Survey was also a cross-sectional, web-based survey with over 80,000 participants from five EU countries [27]. Respondents were members of various online opt-in survey panels; in Italy and Spain online recruitment was supplemented by telephone recruitment among elderly. In that survey 16,340 respondents reported migraine headaches in the past 12 months from which 1680 randomly selected participants filled out the questionnaire on migraine.

The studies using these survey results and documenting migraine-related health care resource utilization are reviewed in Table 2.^4^ Three studies estimate health care resource utilization using the survey data from IBMS-I [28–30]. Bloudek et al. [28] quantify the direct medical cost, excluding medication, in chronic and episodic migraine in five EU countries. Stokes et al. [29] assess health care resource use and related costs, including medication, in chronic and episodic migraine in the USA and Canada. In contrast, Blumenfeld et al. [30] compare episodic to chronic migraine sufferers and investigate whether the headache frequency determine headache-related disability, health-related quality of life and health care resource utilization pooling data from nine countries together. Blumenfeld et al. [30] report health care resource utilization data for the pooled sample and for visits only, whereas Bloudek et al. [28] and Stokes et al. [29] report results both for visits and diagnostic evaluations split by the five EU and two North-American countries, respectively.

**Table 2:** Health care resource utilization in various studies

Study	Methodology	Sample	Migraine type	Health care resource utilization covered	Detailedness of data
Bloudek et al. [28]	Web-based survey in 5 EU countries (IBMS-I). Participants had to report the frequency of visits to various health care professionals occurring over the preceding 3 months for headache treatment or diagnostic evaluation.	5655 (UK 1070; France 1461; Germany 1449; Italy 976; Spain 699)	Participants with CM (*n* = 276) and EM (*n* = 5379) is. CM: ≥15 MHDs; EM: < 15 MHDs.	Primary care physician visits Neurologist/headache specialist visits Nurse practitioner/physician assistant visits Other specialist visits Emergency room visits Hospitalizations Diagnostic testing Blood tests Botulinum toxin A injections Transcutaneous nerve stimulator Acupuncture Occipital nerve block procedures	Proportion of participants reporting one or more visits or diagnostic evaluation among the patients in the category (patients with CM vs EM) in a particular country. Mean number of events of those reporting one or more visits or diagnostic evaluation.
Stokes et al. [29]	Web-based survey in North-America (IBMS-I). Participants had to report the frequency of visits to various health care professionals occurring over the preceding 3 months for headache treatment or diagnostic evaluation. Participants also had to report medications used over the preceding 4 weeks.	1886 (USA 1205; Canada 681)	Participants with CM (*n* = 159) and EM (*n* = 1727) is. CM: ≥15 MHDs; EM: < 15 MHDs	Primary care physician visits Neurologist/headache specialist visits Nurse practitioner/physician assistant visits Other specialist and health professional visits (obstetrician/gynaecologist, pain specialist, psychologist, psychiatrist, social worker) Emergency room visits Hospitalizations Diagnostic tests (MRI, CT, EEG, ECG, X-ray) Blood tests Botulinum toxin A injections Transcutaneous nerve stimulator Acupuncture Occipital nerve block procedures Preventive, acute and other medication	Proportion of participants reporting 0,1, 2 and 3+ visits or diagnostic evaluation among the patients in the category (patients with CM vs EM) in a particular country. Proportion of participants using preventive, acute headache therapy or other medications among the patients in the category (patients with CM vs EM) in a particular country. Mean days of medication usage.
Blumenfeld et al. [30]	Web-based survey in nine developed countries (IBMS-I). Participants had to report the frequency of visits to various health care professionals occurring over the preceding 3 months for headache treatment.	8726 (Australia 516; Canada 681; France 1461; Germany 1449; Italy 975; Spain 701; Taiwan 667; UK 1070; USA 1205)	Participants with CM (*n* = 499) and EM (*n* = 8277) is treated separately. CM: ≥15 MHDs; EM: < 15 MHDs.	Primary care provider visits (including visits to primary care, nurse practitioners, and physician assistant providers) Neurologist/headache specialist visits Emergency department visits Hospital visits Emergency department and hospital visits	Proportion of participants reporting one or more visits among the patients in the category (patients with CM vs EM) in the sample. Mean number of visits of those reporting one or more visits. Data is not split by country.
Sanderson et al. [31]	Web-based survey in six developed countries (IBMS-II). Participants had to report the frequency of visits to various health care professionals occurring over the preceding 3 months for headache treatment. Participants also had to report medications ever tried and currently used.	1165 (USA 431; Canada 105; France 168; UK 157; Germany 193; Australia 113)	Participants with CM (*n* = 493) and EM (*n* = 672) is treated separately. CM: ≥15 MHDs; EM: < 15 MHDs.	Any health care provider visit Have a typical headache care provider Emergency department visit Hospitalization Migraine preventive agents ever tried Migraine preventive currently used Migraine acute agents ever tried Migraine acute currently used	Proportion of participants reporting one or more visits among the patients in the category (patients with CM vs EM) in a particular country. (Mean number of events of those reporting one or more visits is not reported.) Proportion of participants who have ever tried particular number of preventive and acute agents. Proportion of participants who use preventive agents. Type of migraine preventive and acute agents currently used. Medication usage is reported in each category (patients with CM vs EM) in a particular country.
Vo et al. [27]	Data from the 2016 National Health and Wellness Survey is used in 5 EU countries. Migraine respondents were propensity score matched with non-migraine controls. Participants had to report the frequency of visits to various health care professionals in the past 6 months.	218 patients with migraine, 218 patients without migraine. (France 39; Germany 59; UK 67; Italy 31; Spain 22)	Migraine is defined as ≥4 MHDs. Subsamples cover EM (4–7 and 8–14 MHDs) and CM (≥15 MHDs).	Any health care provider visits General/ family practitioner visits Neurologist visits Psychiatrist visits Emergency department visits Hospitalizations	Proportion of participants reporting one or more visits among the patients in the category (patients with vs without migraine) and in the subsamples (4–7, 8–14 and ≥ 15 MHDs). Mean number of visits in the category (patients with vs without migraine) and in the subsamples (4–7, 8–14 and ≥ 15 MHDs). Data is not split by country.
Martelletti et al. [32]	Cross-sectional, multi-country online survey among adults. Oversampling of patients with prophylactic treatment failure defined as change in preventive medication.	11,266 respondents from 31 countries	Migraine is defined as ≥4 MHDs. Subsamples cover migraineurs with one and two or more prophylactic treatment failure.	General practitioner visits Neurologist visits Pharmacist visit Headache specialist visit Dentist visit Physiotherapist visit Psychologist and psychiatrist visit Emergency department visits Hospitalizations Brain scan	Proportion of participants reporting one or more visits. Mean number of visits is reported only for emergency department visits, hospitalizations, and brain scans.

*CM* Chronic migraine, *CT* Computed tomography, *EM* Episodic migraine, *ECG* Electrocardiogram, *EEG* Electroencephalogram, *MHDs* Monthly headache days, *MRI* Magnetic resonance imaging

Sanderson et al. [31] quantify migraine-related health care resource utilization using the survey data from IBMS-II. The authors quantify and compare health resource usage in chronic and episodic migraine across six countries. As compared to IBMS-I, the sample is smaller and covers less European countries. Another major drawback of the study is that it documents only the proportion of participants reporting one or more visits, the mean number of visits is not reported.

From the health care resource utilization data of Vo et al. [27] the incremental consumption of health care resources associated with migraine can be derived. Although the sample in [27] is small (*n* = 218), the estimates are very valuable as they show the incremental usage instead of the migraine-related usage, the approach followed by studies using the survey results from IBMS-I and IBMS-II. Vo et al. [27] report an incremental visit of 2.57, whereas Bloudek et al. [28] find that the total number of migraine-related health care provider visits is 3.17.^5^ This comparison signals that migraine-related health care resource utilization might be slightly overstated in studies without propensity matched controls.

In the recent study of Martelletti et al. [32], the authors describe the disease burden among individuals with migraine for whom preventive treatments failed. The authors administered an online survey in 31 countries worldwide using online bulletin boards. In their sample around 80% of the respondents had a history of prophylactic treatment failure. The authors report higher health care resource utilization (brain scan, emergency department visits, hospital stay) for patients who switched therapies at least two times.

Health care resource utilization estimates in this study are derived from Bloudek et al. [28] for a number of reasons. First, in [28], similar to all other studies using the survey results of IBMS, migreneurs were selected carefully, patients had to meet the ICHD-II diagnostic criteria for migraine. Second, the methodology of IBMS was carefully designed and validated and followed the same approach across the sample countries [25]. Third, the international sample of patients with chronic and episodic migraine is large; as of now the largest and the most recent for Europe. Fourth, it covers five European countries, not just one. Fifth, it delivers estimates both about visiting health professionals and diagnostic evaluations. As a drawback, no patients were surveyed from Central and Eastern Europe and the authors did not document the usage of headache medication.

Health care resource utilization estimates used in this study are shown in Table 3. In the sample of 5655 patients in [28], 4.88% of patients were suffering from chronic migraine, while 95.12% from episodic migraine. We consider this proportion of chronic/episodic migraineurs as a valid estimate for Latvia and Lithuania as well.

**Table 3:** Health resource utilization estimates based on the study of Bloudek et al. [28]

	Chronic migraine (4.88%)	Episodic migraine (95.12%)	All migraine (100%)
Primary care physician visits (%)	54.32%	29.81%	31.01%
*Mean number of visits*	*17.86*	*9.87*	10.26
Neurologist/headache specialist visits (%)	30.42%	9.65%	10.67%
*Mean number of visits*	*8.07*	*6.79*	6.86
Nurse practitioner/physician assistant visits (%)	3.27%	1.82%	1.89%
*Mean number of visits*	*10.89*	*26.19*	25.45
Other specialist visits (%)	23.89%	10.17%	10.84%
*Mean number of visits*	*14.59*	*11.58*	11.73
Emergency room visits (%)	10.16%	5.17%	5.42%
*Mean number of visits*	*11.53*	*7.16*	7.37
Hospitalizations (%)	3.97%	1.91%	2.01%
*Mean length of stay*	*19.03*	*9.95*	10.39
Diagnostic testing (%)	20.30%	9.79%	10.30%
*Mean number of diagnostic tests*	*13.03*	*10.21*	10.35
Blood tests (%)	16.66%	7.09%	7.55%
*Mean number of blood tests*	*10.11*	*6.32*	6.51
Botulinum toxin A injections (%)	1.80%	0.77%	0.82%
*Mean number of injections*	*6.46*	*7.93*	7.85
Transcutaneous nerve stimulator procedures (%)	4.34%	2.07%	2.18%
*Mean number of stimulator procedures*	*38.98*	*38.91*	38.91
Acupuncture (%)	9.06%	4.59%	4.81%
*Mean number of acupuncture*	*23.73*	*16.46*	16.81
Occipital nerve block procedures (%)	3.26%	1.32%	1.42%
*Mean number of nerve block procedures*	*8.70*	*11.87*	11.71

Percentage figures show the proportion of participants reporting one or more visits or diagnostic evaluations. The proportion of participants reporting one or more visits or diagnostic evaluations is calculated as weighted average across the five sample countries. The mean number of visits or diagnostic evaluation of Bloudek et al. [28] was translated into annual figures

#### Reduced workforce participation

Migraine may lead to reduced participation in the labour force through difficulties in obtaining and keeping full-time work. Migraine sufferers face barriers in finding secure, fulltime employment, and the ones being employed are constantly exposed to the fear of losing their jobs because of repeated absences or migraine-induced dysfunction. Generally, migraineurs are suggested to look for flexible jobs, such as writing, graphic design, programming, accounting and the ones that enable remote work [33]. Migraineurs thus may deliberately not enter the labour market as full-time employees or may willing to do so but unable to find and keep their jobs.

Labour force participation of migraine sufferers is lower than in the total working-age population. Empirical evidence shows that the unemployment rate for patients with less severe headache is very similar to the unemployment rate for the average population [4]. Migraineurs with high pain but moderate activity limitations had twice as high unemployment rate as the average population, while migraineurs with severe activity limitations had more than four times as high unemployment rate. Stang et al. [34] find that over the three-year study period, 12% of patients suffering from headache were unemployed, while 13% unable to obtain or keep their full time work due to their condition. The authors identified five factors increasing the likelihood of being unemployed, suffering from migraine being one of them, while being a female, young (aged 18–24), less educated, and having depressive symptoms being the others. Similarly, Stewart et al. [14] also document reduced workforce participation for migraineurs. The authors find that individuals with chronic and high frequency migraine were less likely to be actively working for pay compared with migraineurs with low frequency headache. In particular, the authors report that only 37% of individuals with chronic migraine were employed full time; the respective figure is 48% for migraineurs with less than ten headache days in the past 3 months.

In majority of studies, unemployment is not captured as a component of indirect cost [5, 6, 35]. Unemployed respondents are typically systematically excluded from the analysis; lost workdays are only estimated for the ones being at least part-time employed. Nevertheless, several studies show that workforce participation of migraine sufferers is lower than in the total working-age population [4, 14, 34]. In this study we assess productivity loss as a result of migraine sufferers being unemployed due to their condition. Although people with high frequency headache is less likely to be employed than the general population [4, 14], we conservatively assume that the prevalence rate of migraine among employed and unemployed migraineurs is the same. Moreover, we assume that only individuals with high frequency migraine are unemployed as a result of their migraine. For the remaining migraineurs the primary reason of their unemployment are conditions other than migraine—lower level of education, injury, poor physical or mental health, etc. These assumptions are in line with the findings reported in [4]. Based on Stewart et al. [14], 10.55% of migraineurs is considered as individuals with high frequency migraine.

#### Absenteeism

Table 4 displays the most important findings of population-based studies documenting the number of days missed from work due to migraine identified by targeted literature search. Four inclusion criteria were defined. First, studies shall report the number of days missed from work for patients with migraine in general. Studies were excluded if they documented absenteeism for specific patient groups only, such as migraine with aura or chronic migraine. Second, respondents should be recruited from the general population. Studies were excluded if they were not representative of the entire population. For example, estimates on absenteeism for 723 headache sufferers at a large Swiss university hospital were excluded [38]. The third restriction was related to the geographic coverage of the studies; studies from North-America or Europe were included only. Fourth, studies should be published in the last 10 years (after 31 December 2008), for obtaining up-to-date information and arriving at a reliable, valid estimate. As a result of the targeted literature search, nine studies met the inclusion criteria, these studies are listed in Table 4 and summarized briefly in the following paragraphs.

**Table 4:** Absenteeism days per year

Study	Sample	Episodic migraine	Chronic migraine	All migraine
Munakata et al. [6]^a^	7795 respondents with migraine. Data from the 2006 follow-up survey is used being part of the American Migraine Prevalence and Prevention (AMPP) Study.			1.71
Kessler et al. [36]	3655 employed or self-employed respondents in the US. Part II sample from the National Comorbidity Survey Replication, face-to-face household interviews.			10.7
Stewart et al. [14]^b^	6204 respondents with migraine and active employment status from the US. Data was retrieved from the 2005 American Migraine Prevalence and Prevention (AMPP) study.	2.81	5.20	2.91
Steiner et al. [35]^c^	8271 participants from 9 EU countries (Austria 644; France 876; Germany 318; Italy 487; Lithuania 573; Luxembourg 1833; Netherlands 2414; Spain 999; United Kingdom 127).			12.8
Ayzenberg et al. [7]^d^	2725 adults from Russia (participants aged 18–65 years from 35 cities and nine rural areas; door-to-door survey). Of these, 1273 reported headaches.			0.8
Vo et al. [27]^e^	218 patients with migraine; 2018 patients without migraine (France 39; Germany 59; UK 67; Italy 31; Spain 22).	7.99	25.70	12.55
Vo et al. [37] ^f^	3106 Migraine Buddy© Smartphone users with paid work from 17 European countries.	19.8	52.8	27.6
Martelletti et al. [32]^g^	6534 patients with migraine from 31 countries worldwide being employed full-time or part time.			28.8

^a, b, c, d, e, f, g^ Details of deriving the annual number of days missed from work is reported in Additional file 1

The first three studies shown in Table 4 used data from US patients. Munakata et al. [6] retrieve data from the 2006 follow-up survey part of the AMPP study. The authors document that patients with migraine missed 13.7 h per year from work, which is equivalent to 1.71 missed days per year. Kessler et al. [36] use data from the National Comorbidity Survey Replication. The authors investigate the predictive associations between migraines and workplace outcomes, and whether comorbidity can explain this association. The authors measure absenteeism over the past month with the WHO Health and Work Performance Questionnaire. The authors perform linear regression analysis to evaluate the predictive effects of migraines on absenteeism while controlling for socio-demographic characteristics. They find that migraine is significantly associated with absenteeism; it results in 10.7 excess sickness absence days per year. Stewart et al. [14] report estimates on absenteeism for migraine sufferers participating in the AMPP study in 2005 in the US. Respondents who reported active, severe headache in the screening survey received a second, self-administered headache questionnaire about employment and lost productive time. Out of 11,624 respondents meeting the criteria for migraine and included in the analysis, 6204 were employed actively for pay, either full-time or part-time. The authors document missed hours per actively employed worker per week for four patient groups: low, moderate and high frequency headache, and chronic migraine (< 10 days, 10–29 days, 30–44 days, or > 45 days of headache in 3 months, respectively). Among participants with active employment status, lost productive time was substantially higher for respondents with high frequency headache compared to those with low frequency headache. Migraineurs with 30–44 headache days in the last 3 months had the highest probability to report missed workdays due to headache in the last 2 weeks; they were followed by respondents suffering from chronic migraine. As Stewart et al. [14] report hours per worker per week missed from work, the estimates were converted into annual figures for comparison purposes. Moreover, the four patient groups were merged into two groups (patients with episodic migraine and patients with chronic migraine), for details see the footnote added to Table 4.

Steiner et al. [35] surveyed 8271 participants in 9 EU countries in the Eurolight project with the aim of measuring the personal impact of headache. The authors deliver estimates of lost workdays, housework days and social days due to migraine. The authors report that the impact of migraine is severe; 17.7% of males and 28.0% of females lose more than 10 days of activities (workdays, housework days and social days) in a 3-month period. Due to migraine, the total sample population lost 3.2 working days in preceding 3 months, which translates into losing almost 13 days per year. The figure reported in Table 4 can be considered as a combined absenteeism and presenteeism estimate; it includes both the workdays lost completely and the workdays with productivity reduced to 50% or more of the expected productivity.

Ayzenberg et al. [7] evaluate headache-attributed burden and its impact on productivity and quality of life in Russia. Face-to-face interviews were conducted with 2725 adults in urban and rural areas; the random sample is representative of the population. Headache-attributed lost time results were available for 1273 participants reporting headache. For all headaches, the authors estimate that the mean lost paid-work days in the preceding 3 months were 1.9 days. The authors provide detailed data for the following subsamples: participants with migraine, participants with tension-type headache and participants with headache on 15 days or more (either migraine or tension-type headache). In Table 4 data for respondents with migraine is shown; Ayzenberg et al. [7] report that they missed paid work only on 0.2 days in the preceding 3 months.

Vo et al. [27] provide absenteeism estimates for 218 patients with and without migraine from five European countries. The authors find that respondents with migraine when compared with non-migraine controls reported significantly higher absenteeism, the percentage of work time missed in the past 7 days was 14.43% vs 9.46%, respectively. Based on our calculations, migraine sufferers missed 12.55 days more than the non-migraine controls (see Table 4, footnote).

Vo et al. [37] use 28-day data captured through the Migraine Buddy© Smartphone application from the period of June 2015 – July 2016. Users were self-diagnosed adults from 17 European countries. Data were retrieved for 3900 individuals suffering from migraine; they were selected randomly from a population of 13,032 meeting the inclusion criteria. Of these, 3106 had a work; in Table 4 days missed from work is reported for this subsample. The authors find that migraine attack affected 8.3 days per month an average. In work absenteeism-related attack users most commonly noted body pain, mood and cognition, environmental handicap, depression and/or sleep alterations. Vo et al. [37] thus find that migraineurs miss more than twice as much days from work as the respondents in the Eurolight project [35]. The authors argue that this difference might be related to the study samples. In the sample in [37] more severe patients were included; patients suffered from at least four monthly migraine days and had headache in at least two consecutive weeks from the time of initial registration.

Finally, in a recent study Martelletti et al. [32] report both days missed from work and paid sick days in the last month for over six thousand migraineurs in employment from 31 countries. The authors find that employees suffering from migraine missed an average of 4.6 working days in the last month. The number of paid sick days was significantly smaller, 2.4 days per month. When considering the paid sick days, the findings of Martelletti et al. [32] is comparable to that of Vo et al. [37] . Their sample included more severe patients; they have oversampled patients for whom at least two preventive migraine treatment had failed.

For all migraine patients, the annual number of days being absent from work ranges from the conservative estimate of 0.8 days in [7] to the liberal estimate of 28.8 days in [32]; the latter figure is more than 30 times larger than the former. Had we taken not only the paid sick days as reported in [32] but all the days missed from work, we would arrive at an estimate of 55.2 days missed from work per year. The two highest estimates are derived from a sample of severe migraineurs [32, 37]; these figures are most probably not representative of all migraine sufferers. The remaining estimates are fairly polarized and can be divided into two separate groups. The first group contains estimates ranging from 0.8 to 2.91 days missed from work [6, 7, 14], while the second one consists of estimates ranging from 10.7 to 12.8 days missed from work [27, 35, 36]. Before selecting the absenteeism estimate to be used in this study, we review the relevant literature on presenteeism as well.

#### Presenteeism

Measuring impairment is challenging; researchers must assess whether the disability to think clearly, lack of focus, and loss of concentration result in productivity loss at all, or the work can be performed as usual. If the work cannot be performed as expected, then the number of hours lost shall be calculated. Reduced productivity due to migraine is typically assessed by the Migraine Disability Assessment (MIDAS) questionnaire; participants are asked to report the number of days when their productivity is reduced by half or more [39]. Days when the productivity is reduced by less than half of the expected are ignored.

Table 5 displays the most important findings of population-based studies on productivity loss when working with migraine. The inclusion criteria were the same as for the studies reporting the number of days missed from work due to migraine. As a result of the targeted literature search, four studies met the inclusion criteria, these studies are listed in Table 5.

**Table 5:** Presenteeism days per year

Study	Sample	Episodic migraine	Chronic migraine	All migraine
Munakata et al. [6]^a^	7795 respondents with migraine. Data from the 2006 follow-up survey is used as part of the American Migraine Prevalence and Prevention (AMPP) Study.			6.04
Stewart et al. [14]^b^	6204 respondents with migraine and active employment status from the US. Data was retrieved from the 2005 American Migraine Prevalence and Prevention (AMPP) study, a longitudinal population-based survey.	8.17	24.70	8.90
Ayzenberg et al. [7]^c^	2725 adults from Russia (participants aged 18–65 years from 35 cities and nine rural areas; door-to-door survey). Of these, 1273 reported headaches.			6.8
Vo et al. [27]^d^	218 patients with migraine, (France 39; Germany 59; UK 67; Italy 31; Spain 22	3.52	26.12	9.31

^a, b, c, d^ Details of deriving the annual number of days lost due to impairment is reported in Additional file 2

The first two studies shown in Table 5 used data from US patients, the latter two build upon survey results from Europe. Both studies from the US use data from the AMPP study. Munakata et al. [6] employ the results from the 2006 follow-up survey, whereas Stewart et al. [14] rely on the responses from the 2005 survey. Munakata et al. [6] find that patients with migraine lost 48.3 h per year due to presenteeism, which is equivalent to 6.04 lost workdays per year. Stewart et al. [14] report the hour-equivalent of headache-related reduced performance on days at work for 6204 respondents who were employed actively for pay. Similar to absenteeism, the authors report the hours per worker per week for four patient groups: low, moderate, high frequency headache, and chronic migraine. Patients with chronic migraine had the highest probability to report reduced performance due to headache in the past 2 weeks (3.8 h per week); they were followed by respondents suffering from very frequent headache (2.8 h per week). As Stewart et al. [14] report hour-equivalent of headache-related reduced performance per worker per week, the estimates were converted into annual figures for comparison purposes, for details see Table 5, footnote.

By interviewing Russian adults, Ayzenberg et al. [7] document for participants with migraine that the numbers of days in which productivity was less than 50% of the expected productivity was 1.7 days in preceding 3 month translating into 6.8 days per year (Table 5). Vo et al. [27] provides presenteeism estimates for 218 patients with and without migraine from five European countries. The authors find that respondents with migraine when compared with non-migraine controls reported significantly higher presenteeism, the percentage of impairment while at work in the past 7 days was 35.52% vs 20.97%, respectively. To arrive at the number of days lost due to impairment for patients with migraine, the incremental difference in impairment between patients with migraine and non-migraine controls is multiplied by the number of days working with migraine (for details see Table 5, footnote).

In sum, in recent nation-wide studies the number of days lost due to impairment ranges from 6.04 to 9.27 days per year. This lower range – as compared to the range reported for missed workdays – might be explained by less severe consequences of being present with decreased functional capacity as compared to missing a workday completely; there is no need to submit a medical certificate and employees are less exposed to the fear of losing their jobs.

#### Lost workday estimate of this study

In further analysis, the figure from [35] is used for estimating productivity loss; each year individuals suffering from migraine lose 12.8 workdays due to headache. This estimate includes workdays lost both due to sick leave and impairment; the authors estimate total productive time lost at work as the sum of workdays lost completely due to absenteeism and workdays with productivity reduced to 50% or more of the expected productivity. Steiner and Lipton [40] argue that this approach counterbalances those working days when the productivity was reduced by less than half of the expected, which are ignored otherwise. This approach has already been introduced by MIDAS and was validated in [41].

The estimate of Steiner et al. [35] is considered as a reliable estimate for a number of reasons. First, the authors surveyed over 8000 participants, their sample is the largest. Second, the authors collected data from nine EU countries in a cross-sectional survey. Data collected in the US or Russia might not be valid for Latvia and Lithuania due to the difference in sick leave regulation, among others. Although the sick pay and sickness benefit schemes are not harmonized in the European Union, they share several common characteristics [42]. Steiner et al. [35] show that personal impact is terms of lost useful time was surprisingly uniform across the sample countries. Third, the authors employ an already validated method being widely used for assessing the impact of headache.

The 12.8 days productive time lost at work estimate from [35] for Europe is comparable to the findings from [14] for the US. As shown in Tables 4 and 5, Stewart et al. [14] document that migraine sufferers are absent from work on 2.9 days and lose additional 8.9 workdays due to reduced productivity, resulting in 11.8 lost workdays in total.

### Data retrieved from Latvia and Lithuania

#### Number of migraineurs

In order to estimate the economic cost of migraine in Latvia and Lithuania, the number of individuals suffering from migraine should be determined. Prevalence rates are typically derived for the adult population [18–20]; prevalence rates for children and elderly are typically much smaller [3]. As a result, the number of people aged 18–65 is taken from both Latvia and Lithuania. As of 1 January 2017, in Latvia 1,228,779 people, whereas in Lithuania 1,819,685 people were aged 18–65 [43]. By assuming a prevalence rate of 11.4% as reported in [18], the number of individuals suffering from migraine is 140,081 in Latvia, and 207,444 in Lithuania.

#### Unit costs related to health care resource utilization

Unit costs were derived from several national data sources in Latvia and Lithuania. For Latvia, unless indicated otherwise, information about publicly funded medical care was retrieved from [44]. For Lithuania, unless indicated otherwise, information about publicly funded medical care was retrieved from [45]. In Latvia, several medical services are provided by both publicly and privately funded providers. In the most conservative scenario (labelled as the base case), unit cost of publicly funded services is preferred over privately funded services. As we aim to access the total cost of migraine for the society regardless of the payer, patient co-payment, if present, is included in the unit cost. The unit cost of various health care services and procedures is shown in Table 6; details about estimating the unit cost of one visit in primary care, and the split between public funding and patient co-payment is reported in Additional file 3.

**Table 6:** Unit cost of various health care services and procedures in Latvia and Lithuania

	Latvia - public funding	Latvia - private funding	Lithuania (this study)	Lithuania [5]
Primary care physician visit	4.67^a^	28.46^a^	4.49^b^	2.9
Neurologist/headache specialist visit	18.31^c^	40^c^	26.11^d^	24
Nurse practitioner/physician assistant visit	4.77	4.77	NA	NA
Other specialist visit (average)				29
*Obstetrician/gynaecologist*	13.92^e^	30^e^	15.16^f^	
*Pain specialist*	15.31^g^	40^g^	26.11^h^	
*Psychologist*	35^i^	35	3.42^j^	
*Psychiatrist*	8.61^k^	35^k^	21.68^l^	
Emergency room visit	38.79^m^	117.57^m^	26.11^n^	19.4
Hospitalization (per case or per day, as indicated)	93.7^o^	93.7^o^	163.5^p^	89.9^q^
Diagnostic testing				
*MRI brain/scan*	112.82^r^	115^r^	125.4	103.8
*CT brain/scan*	25.65^s^	93^s^	66.79	55.3
*Electroencephalogram (EEG)*	25.44^t^	42^t^	21.68^u^	NA
*Electrocardiogram (ECG)*	6.14	12^v^	15.16^w^	NA
*X-ray neck/scan*	13.50^x^	14.85^x^	15.16^y^	32.4
Blood test	3.05^z^	5.75^z^	7.5^α^	2.9
Botulinum toxin A injection	495^β, γ^	495^β, γ^	450^β^	NA
Transcutaneous nerve stimulator procedure	NA	NA	NA	NA
Acupuncture	52.9^β, γ^	52.9^β^	20^δ^	NA
Occipital nerve block procedure	24^β, ε^	24^β, ε^	NA^ζ^	NA
Medication	12.26 ^η^	21.30^θ^	9.39 ^ι^	6.32

^a, b, c, d, e, f, g, h, i, j, k, l, m, n, o, p, q, r, s, t, u, v, w, x, y, z, α, β, γ, δ, ε, ζ, η, θ, ι^ See Additional file 3 for further details. NA = Not available

Estimating the medication cost for patients with migraine is challenging. Some medications are listed explicitly as migraine medications; they could be identified from administrative databases by drug or disease classification codes. Nevertheless, several over-the-counter analgesic drugs are used to treat migraine; these pain killers, such as paracetamol, aspirin, and ibuprofen have a wide array of applications; the ones used for migraine cannot be easily extracted from administrative databases.

As a conservative approach, we estimate medication costs by calculating the annual cost of typical acute migraine therapies and choosing the cheapest therapy. Additional file 4 lists the annual cost of acute migraine therapies in Latvia and Lithuania by assuming monthly 4 headache days. Annual cost of therapies with paracetamol, nonsteroidal anti-inflammatory drugs (NSAIDs) such as ibuprofen and aspirin, and triptans are calculated; these typical therapies were identified from [46]. We assume that paracetamol, ibuprofen and aspirin are taken on each day with headache, while triptans are taken once per attack, assuming monthly one attack. As shown in Additional file 4, in general, therapies with ibuprofen are the cheapest, followed by paracetamol and aspirin; treatments with triptans are much more expensive. In the absence of detailed data on medication usage by patients, we assume that patients with high frequency headache take the cheapest available triptans, whereas all other patients use the cheapest over-the-counter analgesics, both in the defined daily dose. Based on Stewart et al. [14], 10.55% of migraineurs is considered as individuals with high frequency migraine—migraine on more than 10 days per month, including chronic migraine. The assumption that 10.55% of patients use triptans once per month can be considered as a conservative assumption; in North-America 16.8% of patients with migraine use triptans on average on 4.75 days in the past month [29].

As shown in the last row of Table 6, for Latvia we arrive at an annual mean per patient medication cost of €12.26, derived as (89.45% *×* 10.21) + (10.55% *x* €29.67), where 89.45% is the proportion of patients with low and moderate headache frequency as reported in [14], €10.21 is the annual cost of the cheapest therapy with analgesics (Additional file 4), 10.55% is the proportion of patients with high headache frequency as reported in [14], and €29.67 is the annual cost of the cheapest therapy with triptans (Additional file 4). For Lithuania, we arrive at an annual mean per patient medication cost of €9.39, derived as (89.45% *x* €9.21) + (10.55% *x* €10.83), following the same logic as for Latvia. A sensitivity test with an alternative medication cost estimate is then performed. We also discuss the validity of our medication cost estimate and we compare it with estimates from other European countries.

In sensitivity test unit cost of privately funded services in Latvia is used (Table 6). Latvian patients frequently visit private providers due to significantly shorter waiting times. The fee of privately funded health care services varies greatly, see Additional file 3. In sensitivity test the lowest reported fee of the first visit is inserted for private consultations. Additional details about the unit costs used in the sensitivity test is reported in Additional file 3. For example, the fee of nurse practitioner/physician assistant visit and hospitalizations remain unchanged, whereas the fee of emergency room visit almost triples. Fees also remain unchanged if the entire fee is paid out of pocket (e.g., transcutaneous nerve stimulator procedure, acupuncture). In contrast, visiting a primary care physician in private setting is six times more expensive than a publicly funded visit. Similarly, fees of consultations with specialists and several diagnostic evaluations are much higher in private setting as compared to public setting (e.g., neurologist visit, pain specialist visit, brain CT). For medications we insert €21.30; related assumptions are reported when performing the sensitivity tests.

We have also carried out a systematic search in Pubmed to identify any previous unit cost estimates, for comparison purposes. As unit prices differ highly across countries, we only consider the estimates reported for Latvia and Lithuania as relevant.^6^ The systematic search yielded one relevant study, which provides unit prices for eight European countries, including Lithuania [5]. Their estimates for Lithuania are shown in the last column of Table 6; the estimates have not been adjusted by inflation and thus allow only rough comparison.

#### Employment-related data

Several employment related data is retrieved from Latvian and Lithuanian databases; from the databases of the Central Statistical Bureau of Latvia and Statistics Lithuania, principal government institutions in charge of statistics and census data. The number of registered unemployed persons is required to estimate the productivity loss related to reduced workforce participation. As for unemployed migraineurs we consider that the entire annual gross income is lost, information about the average annual gross wages and salaries of employees in Latvia and Lithuania is also retrieved. Finally, for estimating the loss incurred through absenteeism and presenteeism, information about the average daily income and number of workdays in 2017 in Latvia and Lithuania is collected.

## Results

### Total cost related to health care resource utilization

Total cost related to health care resource utilization is estimated as the product of three components:

1. Proportion of patients reporting one or more visits or diagnostic evaluations as reported in [28]; see figures expressed in percentages in the last column of Table 3.
2. The mean number of visits or diagnostic evaluations for patients reporting at least one visit, as reported in [28]; see figures expressed in digits in the last column of Table 3.
3. Unit cost price as reported in Table 6.

As no information is available about the proportion of patients visiting other specialists, such as obstetrician/gynaecologist, pain specialist, psychologist and psychiatrist, we assume equal distribution across these specialists. Similarly, no information is available about the proportion of patients with various diagnostic tests, such as brain MRI, brain CT, EEG, ECG and neck X-ray. For diagnostic tests, we assume that these diagnostic tests are performed proportionally in the same way as reported in [47]. Based on [47], for example, we assume that CT brain/scan is used almost twice as often as X-ray, while MRI is used roughly half as often as X-ray.

Unit cost was considered as zero for transcutaneous nerve stimulator procedure both in Latvia and in Lithuania; this procedure is not offered to patients. Unit cost was considered as zero for visits to nurse practitioner/physician assistant in Lithuania; this system is non-existent there. Similarly, unit cost was considered as zero for the occipital nerve block procedure in Lithuania; this procedure is performed together with botulinum toxin A injections and is included in its fee to be paid out-of-pocket.

Table 7 displays the annual per person direct cost for patients with migraine in Latvia and Lithuania, assuming publicly funded services where available. Mean per-person annual costs of migraine is €205.77 in Latvia, and €177.73 in Lithuania. The most significant costs are incurred as a result of diagnostic testing, visiting various doctors, such as primary care physicians, neurologists, pain specialists, and receiving botulinum toxin A injections. Table 7 shows the total direct cost per annum by assuming a migraine prevalence rate of 11.40% estimated in [18]. In Latvia total cost of migraine-related health care resource utilization is €28.83 million, corresponding to 0.11% of Latvia’s GDP in 2017 [48]. In Lithuania total cost of migraine-related health care resource utilization is €36.87 million, corresponding to 0.09% of Lithuania’s GDP in 2017 [49].

**Table 7:** Direct cost of migraine in Latvia and Lithuania

	Annual per person cost		Annual total cost, thousand EUR	
	Latvia	Lithuania	Latvia	Lithuania
Primary care physician visit	14.85	14.28	2081	2963
Neurologist/headache specialist visit	13.39	19.09	1876	3961
Nurse practitioner/physician assistant visit	2.30	0.00	322	0
Other specialist visit	23.16	21.10	3244	4377
Emergency room visit	15.48	10.42	2169	2162
Hospitalization	19.53	13.12^a^	2736	2722
Diagnostic testing	24.61	41.42	3447	8592
Blood test	1.50	3.69	210	765
Botulinum toxin A injection	31.96	29.06	4478	6028
Transcutaneous nerve stimulator procedure	–	–	–	–
Acupuncture	42.74	16.16	5987	3352
Occipital nerve block procedure	3.98	0.00	558	0
Medication	12.26	9.39	1718	1948
Total	205.77	177.73	28,825	36,869

^a^ As Lithuanian unit cost data is available per hospitalizations, the number of treatment cases should be derived. We conservatively assume that hospitalized patients had only one treatment case in three months. To arrive at an annual treatment case estimate, we take four times the quarterly figure. In each quarter hospitalized patients may or may not overlap with patients hospitalized in previous quarter; it does not influence the cost estimate

We also test the sensitivity of our results to various assumptions. Most importantly, we will use a higher migraine prevalence rate; 12.71% from the Eurolight project for Europe [19]. Moreover, for Latvia, the total cost of migraine-related health care resource utilization will be assessed by replacing the fees of publicly funded services with unit costs of privately funded services.

### Total productivity loss

Per person and total productivity loss is displayed in Table 8. Average annual gross income is considered as the per person loss incurred through *unemployment*. In Latvia, the average annual gross wages and salaries of employees in 2017 was EUR 11,111 [50]. Although people with migraine is less likely to be employed than the general population [14], by adopting a very conservative approach, we assume that among unemployed the prevalence of migraine is the same as among employed. By the end of 2017, there were 63,121 registered unemployed persons in Latvia [51], 11.4% of whom are considered as individuals suffering from migraine, resulting in 7169 unemployed migraineurs [18]. From these unemployed migraineurs only individuals with high frequency migraine are considered as unemployed due to their migraine. Stewart et al. [14] document that 10.55% of migraineurs is suffering from high frequency migraine, migraine on more than 10 days per month. Therefore, there are 756 migraineurs who are unemployed as a result of their headaches. Thus, in Latvia the total productivity loss incurred through unemployed migraine sufferers is €8.43 million, corresponding to 0.03% of Latvia’s GDP in 2017 [48].

**Table 8:** Total productivity loss due to migraine

	Latvia	Lithuania
Adults (aged 18–65) with migraine	140,081	207,444
*Unemployed*	7196	11,753
*of which unemployed due to migraine (10.55%)*	759	1240
*Employed*	132,885	195,691
Cost, unemployed (per person, EUR)	11,111	10,085
Cost, employed (per person, EUR)	564.37	512.25
Total cost, unemployed (million EUR)	8.43	12.51
Total cost, employed (million EUR)	75.00	100.24
Total productivity cost (million EUR) (employed & unemployed)	83.43	112.75
Total productivity cost (in % of GDP)	0.31%	0.27%

In Lithuania, the average annual gross wages and salaries of employees in 2017 was EUR 10,085 [52]. By the end of 2017, there were 103,100 registered unemployed persons in Lithuania [53], 11.4% of whom are considered as individuals suffering from migraine, resulting in 11,753 unemployed migraineurs. 10.55% of these migraineurs is considered as unemployed due to their migraine condition; 1240 individuals in total. As a result, in Lithuania the total productivity loss incurred through unemployed migraine sufferers is €12.51 million, corresponding to 0.03% of Lithuania’s GDP in 2017 [49].

Per person loss incurred through *absenteeism* and *presenteeism* is derived as a product of the annual number of days lost and average daily income. The average daily income is the ratio of the average annual gross wages and salaries of employees in 2017 and the number of working days in 2017. In Latvia, the average daily income was €44.09 in 2017 [50, 54]. In Lithuania, the average daily income was €40.02 in 2017 [52, 54]. Both in Latvia and Lithuania the number of working days in 2017 was 252 [54]. As a result, per person annual productivity loss due to absenteeism and presenteeism is €564.37 in Latvia, while €512.25 in Lithuania. In total, €75.00 million is lost in production due to absenteeism and presenteeism in Latvia, corresponding to 0.28% of Latvia’s GDP in 2017 [48]. The respective figures are €100.24 million and 0.24% for Lithuania. Total productivity cost, considering both employed and unemployed migraineurs, is €83.43 million in Latvia, and €112.75 million in Lithuania.

### Total cost of migraine

**Fig. 2:**
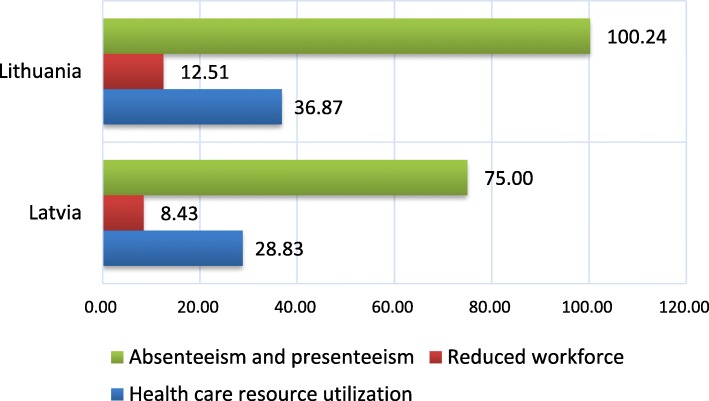
Economic cost of migraine in Latvia and Lithuania in million EUR

Figure 2 summarizes the total cost of migraine, including both direct and indirect costs, for both Latvia and Lithuania in the base case, in the most conservative scenario (see sensitivity test). As shown in the figure, absenteeism and presenteeism accounts for the most significant portion of economic cost (67% in both countries), followed by health care resource utilization, and then by reduced workforce participation.

Table 9 displays the mean per-person total cost of migraine in Latvia and Lithuania. The mean per-person total cost of migraine is €801.37 annually in Latvia, and €721.24 in Lithuania. The mean per-person direct cost is €205.77 annually in Latvia and €177.73 in Lithuania. In Latvia, the mean per-person indirect cost, averaged across employed and unemployed, accounts for €595.59 of which €60.22 was attributable to reduced workforce participation and €535.38 to lost workdays. In Lithuania, the mean per-person indirect cost, averaged across employed and unemployed, accounts for €543.51 of which €60.28 was attributable to reduced workforce participation and €483.23 to lost workdays.

**Table 9:** Per person total productivity loss due to migraine

	Latvia	Lithuania
Direct cost		
*Health care resource utilization*	205.77	177.73
Indirect cost (total)	595.59	543.51
*Reduced workforce*	60.22	60.28
*Absenteeism and presenteeism*	535.38	483.23
Total cost	801.37	721.24

Note that in Latvia the per person annual indirect cost is €11,111 for unemployed and €564.37 for employed (Table 8). In Lithuania the per person annual indirect cost is €10,085 for unemployed and €521.25 for employed (Table 8). In Table 9, mean per-person indirect cost is averaged across all migraineurs, regardless of their employment status.

Table 10 shows the results from three deterministic, univariate sensitivity tests [55]. In the first sensitivity test we use the migraine prevalence rate of 12.71% from the Eurolight project for Europe [19]. This prevalence estimate is based on surveying 9247 individuals in 10 EU countries. This estimate can be considered as a conservative estimate as well; several studies reported a higher prevalence rate (Table 1). As shown in panel B of Table 10, the total cost of migraine increases by 11.49% in both countries as compared to the base case.

**Table 10:** Sensitivity tests

	Latvia		Lithuania	
	in million EUR	in % of GDP	in million EUR	in % of GDP
Panel A: Base case (the most conservative estimate)				
Health care resource utilization	28.83	0.11%	36.87	0.09%
Reduced workforce	8.43	0.03%	12.51	0.03%
Absenteeism and presenteeism	75.00	0.28%	100.24	0.24%
Total cost	112.26	0.42%	149.62	0.35%
Panel B: Prevalence rate of 12.71% from Katsarava et al. [19]				
Health care resource utilization	32.14	0.12%	41.11	0.10%
Reduced workforce	9.40	0.03%	13.94	0.03%
Absenteeism and presenteeism	83.61	0.31%	111.76	0.26%
Total cost	125.16	0.46%	166.81	0.40%
Panel C: Utilization of privately funded health services in Latvia				
Health care resource utilization	54.00	0.20%	36.87	0.09%
Reduced workforce	8.43	0.03%	12.51	0.03%
Absenteeism and presenteeism	75.00	0.28%	100.24	0.24%
Total cost	137.44	0.51%	149.62	0.35%
Panel D: Absenteeism and presenteeism estimate from Vo et al. [27]				
Health care resource utilization	28.83	0.11%	36.87	0.09%
Reduced workforce	8.43	0.03%	12.51	0.03%
Absenteeism	73.53	0.27%	98.29	0.23%
Presenteeism	54.55	0.20%	72.91	0.17%
Total cost	165.34	0.61%	220.57	0.52%

In the second sensitivity test the total cost of migraine-related health care resource utilization in Latvia is assessed by replacing the fees of publicly funded services with unit costs of privately funded services. In this scenario we use the annual mean per patient medication cost of €21.30 as reported in Table 6. This estimate is derived by assuming that 67.6% of the patients take analgesics (paracetamol), 48.0% use NSAIDs, whereas 16.8% are treated with triptans, as reported for US and Canadian migraineurs [29]. Conservatively, we assume that analgesics and NSAIDS are taken 4 days per month, whereas triptans are taken once per attack, assuming monthly one attack, both in the defined daily dose. Stokes at al. [29] report that patients use these medications with much higher frequency per month. In each case the cheapest available therapy is considered, as reported in Additional file 4. Mean per-person annual costs of migraine increases from €205.77 to €385.52. The total cost of migraine-related health care resource utilization increases to €54.00 million, by almost 90% (Table 10, panel C).

In the third sensitivity test we employ the absenteeism and presenteeism estimates from [27] (Table 10, panel D). Although the sample of the authors is small (*n* = 218), they provide absenteeism and presenteeism estimates for European patients both with and without migraine, from which the incremental difference between the two groups can be derived. This incremental difference can be considered as days missed due to migraine. Vo et al. [27] report that respondents with migraine missed 12.55 days more from work when compared with non-migraine controls, while due to impairment they could not work on 9.31 additional days. In comparison with the base case, total cost of migraine increases significantly, by 47.29% in Latvia and 47.42% in Lithuania. In this scenario, in Latvia the total cost of migraine is €165.34 million, corresponding to 0.61% of Latvia’s GDP in 2017 [48]. In Lithuania the total cost of migraine is €220.57 million, corresponding to 0.52% of Lithuania’s GDP in 2017 [49].

## Discussion

### Comparison with previous estimates

Some recent European studies report per person mean annual and/or total cost of migraine. In the following we compare our findings with those cost estimates. In the Eurolight project Linde et al. [5] find that the mean per-person annual cost of migraine in the eight sample countries is €1222. The authors report that direct costs accounted for only 7%; the most important cost element was outpatient care (€30), followed by medical investigations (€19), acute medications (€16), hospitalization (€16) and prophylactics (€5). Indirect costs accounted for 93% of total costs, of which one-third was attributable to absenteeism (€371), and two-thirds were related to reduced productivity (€765). From the eight sample countries in the Eurolight project Lithuania was the only one from Central and Eastern Europe. For Lithuania, the authors document that the mean per-person annual cost of migraine is €297, less than one-fourth of the cost reported for the pooled sample [5]. Direct cost accounted for €54.9 corresponding to 18.47% of total cost; the most important cost element were diagnostic investigations (€20.3) and hospitalizations (€19.6), followed by outpatient care (€8.7) and acute medications (€6.3). Indirect costs accounted for €242.4 equalling to 81.53% of total costs, of which €56.6 was attributable to absenteeism, and €185.9 to reduced productivity. In this study we document much higher mean per-person annual total cost of migraine in both Baltic countries: €801 in Latvia, and €721 in Lithuania. Mean per-person direct cost accounted for €206 in Latvia corresponding to 25.68% of total cost. Mean per-person direct cost accounted for €178 in Lithuania corresponding to 24.64% of total cost. In Latvia, mean per-person indirect costs, averaged across employed and unemployed, accounted for €596, of which €60 was attributable to reduced workforce participation and €535 to lost workdays. In Lithuania, mean per-person indirect costs, averaged across employed and unemployed, accounted for €544 of which €60 was attributable to reduced workforce participation and €483 to lost workdays (Table 9).

In the base case, for Latvia we arrived at a mean per patient medication cost estimate of €12.26, whereas in the sensitivity test we used a medication cost estimate of €21.30. In the base case, for Lithuania we estimated the mean per patient medication cost to be €9.39. These estimates are comparable to the ones reported in [5] for several European countries in the Eurolight project. Across all sample countries the medication cost was documented to be €21, including both acute and prophylactic medications. Medication costs varied highly across countries, ranging from €6.32 for Lithuania to €50 for Spain. Moreover, when calculating the total cost of triptan in Lithuania we arrive at an estimate of €278,018.^7^ This estimate is very close to the total cost of reimbursed triptans (€282,255 in 2017, €245,594 in 2018), which validates our estimate.

For Europe, previous studies unambiguously document that indirect cost constitutes the greater part, approximately 70–80% of total cost [5, 56, 57]. This study is in concordance with this split; in Latvia 74.32%, while in Lithuania 75.36% of total cost is indirect cost.

No previous studies measured the total cost of migraine in Latvia. For Lithuania, the total cost of migraine reported in this study is in line with the total cost estimate in [5]. Linde et al. [5] find that the total cost of migraine in Lithuania is €139.74 million, derived as the product of the per person mean annual cost and the number of migraine sufferers (*N* = 469,998). In this study, the respective figure, excluding reduced workforce participation for comparison purposes, is almost the same, €137.11 million (Fig. 2, Table 10, panel A). Nevertheless, in [5] the much higher prevalence rate of migraine is coupled with lower per patient cost estimates. In [5] the much higher prevalence rate can be explained by taking both migraine and probable migraine into account.

Ayzenberg et al. [7], the only relevant study from the Eastern bloc, estimate the indirect costs of primary headache disorders in Russia. By extrapolating the survey results to the employed population, the authors report that indirect cost of migraine amount to USD 7.7 billion per year, or 0.59% of GDP. The indirect cost estimate of this study is lower than the estimate in [7]; in Latvia 0.31% of GDP, while in Lithuania 0.27% of the GDP is missed due to lost workdays. The difference can be explained by considerably higher prevalence rate of migraine in Russia than the global averages used in this study (20.3% vs 11.40%).

### Policy implications

The economic cost of migraine in Latvia and Lithuania is large; the financial burden imposed on both countries is substantial. Although in the absence of cost-benefit analysis it is too early to formulate relevant policy recommendations, we suggest a few future directions worth addressing.

First, awareness about the large economic burden of migraine should be raised among health policy makers and the general population—this research can be considered as a first step towards raising awareness.

Second, patients suffering from frequent headaches should be reached and structured headache assessment services should be offered to them; this service should preferably be based in primary care due it its wider and easier accessibility, and lower costs as compared to specialist services. Steiner et al. [58] provide detailed recommendations on how headache services should be organised and delivered in Europe. In general, primary care should serve as first point of consultation, and general practitioners should offer patients basic acute and preventive treatment. The lower number of monthly headache days as a result of such treatment would then translate into significant economic benefits; the productivity loss will be lower.

Third, access to specialist services should be provided for patients with more severe or complex symptoms—assess which is quick and easy. Migraine sufferers should not only be reached, but effective therapies should also be offered to them. Although several options are available for treating acute migraine attacks, a particular therapy might work for one patient while fail for others. In specialist care, special attention should be devoted to patients suffering from high frequency and intense headaches. Due to reduced workforce participation, absenteeism and presenteeism, these patients are responsible for a significant portion of total costs. Offering preventive, prophylactic medication to patients suffering from frequent and intense migraine attacks might reduce the number of lost workdays significantly. At the same time, by using prophylactic medications patients could avoid medication overuse headache—headache occurring when simple analgesics or triptans are taken frequently to relieve headaches. The reduction in lost workdays and thus the increase in productivity might well exceed the increased health resource utilization and medication costs. In general, had migraineurs suffer from less frequent and intense headaches, would the entire nation benefit from it as a result of increased productivity.

### Methodological considerations

In this study we used the prevalence method to estimate the total cost of migraine. In this approach the total cost estimate is derived as a product of the prevalence rate and mean per person annual cost. For both components, we relied on several *estimates from the literature* (Fig. 1). Instead of the literature we could have derived these figures from surveys carried out in Latvia and Lithuania. Conducting surveys are not only time-consuming and costly, but in addition to careful design and implementation they also require large sample sizes. To increase the reliably and validity of such estimates, the sample size should preferably be larger than what is currently available in the literature. In the absence of resources to conduct such surveys, estimates are drawn from the literature whose advantages and disadvantages are discussed in the following.

First, we used a *migraine prevalence rate* of 11.4% derived for Europe by Woldeamanuel and Cowan [18] in their meta-analysis. The authors pooled data from 140 European studies involving a combined sample size of over 800 thousand participants, corresponding to a sampling fraction of 0.1%. The strengths of this recent meta-analysis include its extensive nature and its methodology; it delivers a prevalence rate weighted by the sample sizes instead of a simple arithmetic average estimate. Nevertheless, similar to all meta-analyses, this estimate might be biased as it is affected by the heterogeneity of the reviewed studies; the definition and measurement of migraine, the prevalence period, the sample size and year of sampling varied across the studies. To overcome such bias, in sensitivity tests we employed a migraine prevalence rate of 12.71% from the Eurolight project as well [19]. Although in the Eurolight project the sampling fraction is much smaller than 0.1%, a strict definition of migraine (migraine on more than 5 days/month) is employed and homogeneous measurement across the 10 EU countries is assured.

Second, the *health care resource utilization* estimates are drawn from the survey results of IBMS characterized by careful identification and selection of patients with migraine, validated methodology and large sample. In particular, we use the estimates from [28] covering 5655 respondents from five Western European countries (Table 2). Health care resource utilization in Central and Eastern Europe, however, might be different from the one documented in Western Europe. For example, the number of self-reported consultations of medical professionals is lower in Latvia and Lithuania than in France, Germany and Italy, similar to Spain and slightly higher than in the UK [59].

Third, as we have no information from surveys on how much migraine sufferers exactly spend on medications relieving their pain, we relied on a number of assumptions to arrive at a *mean per patient medication cost estimate* for Latvia and Lithuania. In the base case, we assumed that 10.55% of the patients take triptans, whereas 89.45% of migraine sufferers use the cheapest over-the-counter analgesics. In sensitivity test, we assumed that 16.8% of the patients are treated with triptans, 67.6% of the patients use paracetamol, whereas 48.0% use NSAIDs, based on survey data reported in [29]. In both cases, we assumed that over-the-counter analgesics (paracetamol, NSAIDs) are taken 4 days per month, whereas triptans are taken once per attack, assuming monthly one attacks, in the defined daily dose. Both estimates can be considered as a rather conservative estimate for several reasons. First, migraine sufferers typically use more than two classes of medications at the same time [29]. Second, a typical patient with episodic migraine takes simple analgesics on 5.4 days in the US and 7.4 days per month in Canada, combination analgesics on 5.75 and 6.2 days, and NSAIDs on 6.9 and 6.6 days, respectively [29]. Patients with episodic migraine use triptans on approximately 4 days per month, whereas patients with chronic migraine use triptans on almost 10 days per month [29]. Third, we have taken the cheapest available therapy from a particular class; patients might not discover prices that consciously. Fourth, not each medication from the same class is equally efficient for each patient. Patients typically need to test several triptans before they can identify the one that works for them, and many times treatment need to be repeated in 48 h [60].

Fourth, when estimating the indirect cost incurred through *reduced workforce*, we conservatively assumed that individuals with migraine are employed with the same probability as the general population. In severely affected migraine sufferers, however, the unemployment rate is found to be significantly higher than in the general population [14, 61]. In Latvia, over 40% of unemployed individuals left their last job due personal or family responsibilities, own illness or disability [62]. In the absence of more detailed reasons for unemployment, we assumed that only individuals with high frequency migraine are unemployed as a result of their migraine. For the remaining migraineurs the primary reason of their unemployment are conditions other than migraine—lower level of education, injury, poor physical or mental health, etc. Alternatively, we might have relied on Stewart et al. [14] reporting that individuals with episodic migraine are 2.8% less likely to be employed than the general population, while individuals with chronic migraine are 19% less likely to be employed than the general population. With these assumptions the number of migraine sufferers being unemployed due to their condition would have been 390 in Latvia and 456 in Lithuania; lower than the ones reported in Table 8.^8^

Fifth, *lost workdays*, either due to absence or impairment, were adopted form [35]; each year individuals suffering from migraine lose 12.8 workdays due to headache. Steiner et al. [35] considered workdays with productivity reduced to 50% or more of the expected productivity as days fully lost. This validated approach counterbalances those working days when the productivity was reduced by less than half of the expected, which are ignored otherwise [40, 41]. As personal impact in terms of lost useful time was surprisingly uniform across European countries [35], had we surveyed individuals suffering from migraine in Latvia and Lithuania might we arrived at very similar estimates. In the literature, the number of days lost due to absenteeism and presenteeism vary greatly, some studies report higher, while others lower number of lost workdays, mostly depending on the definition of patients with migraine (Tables 4 and 5). The sample in [35] includes patients with less severe conditions, patients with migraine and probable migraine were considered together. As a result, their lost workdays estimate can be considered as a conservative estimate; patients with probable migraine, constituting almost 40% of respondents with migraine, highly likely miss less workdays than patients with definite migraine.

### Limitations

The burden of migraine imposed on the society is substantial. In this study we measured the financial burden in two domains, health care resource utilization and productivity loss. It did not capture the constraints imposed on household work, social life and leisure activities; is more difficult to translate these intangible costs into monetary units. We should not nevertheless underestimate the demolishing impact of headache on the migraineurs’ and their family members and friends. Several authors report that migraine sufferers are unable to perform household work on a couple of days per month and miss regularly family, social and leisure activities due to their condition [7, 35]. Migraineurs might even miss more days from family and leisure activities than from work or school [10].

As a second limitation, we did not look at the entire population with migraine. Migraineurs with less than four monthly headache days were disregarded in this study. Therefore, the total economic burden associated with migraine might be underestimated.

Third, we did not consider the economic consequence of underemployed migraineurs in this study. One typical form of underemployment is when a worker is under-used in his part-time job despite longing for full-time work [63]. Several flexible jobs suggested for migraineurs assume freelancers, self-employed workers who are hired for particular projects [33]. Unless a freelancer is a very demanded worker, underemployment is highly probable. Lost career advancement and early retirement can be also considered as special forms of underemployment; the costs related to these forms of underemployment were also not captured in this study. Underemployed migraineurs, either as part-time workers or early retired, might also generate significant productivity losses which was not captured in this study.

Fourth, we have no information whether unemployed migraineurs are actively searching for employment at all. We also do not know whether unemployed migraineurs are unable to find work due to their condition. It might well be the case that they are unable to find work due to their lower level of education, outdated expertise, their ethnicity or disability other than migraine. We have used a very conservative estimate for migraine related unemployment; we assumed that only 10.55% of unemployed migraineurs, the ones with high frequency headache, are unemployed as a result of their condition [14].

Fifth, the majority of cost estimates were retrieved either from publicly available databases or from health care service providers. Per person direct cost estimates are subject to variation in the unit cost estimates. The difference in unit cost estimates is remarkable when fees of publicly funded and privately funded health care services are compared. For example, in Latvia visiting a private neurologist is twice as more expensive as visiting a publicly funded neurologist, whereas a brain CT at a private health care provider costs 3.6 times more than at a public provider. In the absence of information about the proportion of publicly and privately funded medical services and procedures, we conservatively assumed that all visits and procedures are publicly funded. In sensitivity test we looked at the other extreme, and assumed that all visits and medical procedures are privately funded. The reality shall be in between the two extremes.

Sixth, as we have no readily available information on how much migraine sufferers spent on medications relieving their pain, we relied on a number of assumptions and delivered a conservative estimate. Unless a survey among migraineurs is carried out, we cannot validate the annual mean per patient medication cost estimate used in this study.

Seventh, the health care resource utilization data was adopted from voluntary online survey results of the IBMS. In case of voluntary surveys selection bias towards more severe migraineurs is introduced; those with heavy migraine-type headache are more inclined to fill out the questionnaire. As argued in [28], the relatively high proportion of respondents using opioids suggests the selection of migraineurs with more frequent and/or more severe headaches. Moreover, recall bias is introduced; respondents may not be able to remember the number of migraine-related visits properly or may systematically underreport the utilization of particular health care resources to perceive their health status more favourable. Most probably, recall bias affect typical events such as visiting a primary care provider or neurologist. The recall bias shall be considered as minimal for rare events such as hospitalizations, unless a respondent wants to disguise the severity of its migraine which is considered as highly unlikely in voluntarily, anonymous surveys.

Finally, several cost elements were disregarded in this study. For example, we estimated neither migraine-related health administration costs nor research expenditure in the field. Similarly, the costs of several treatments and preventive therapies such as yoga, relaxation, exercise, massage, aroma-therapy mostly paid out-of-pocket, were also ignored. Not only vary their usage highly across countries, but the evidence about their effectiveness is also limited.

Future research might aim at refining the estimates we have derived from the literature. On the one hand, the general population should be surveyed for precising the prevalence rate of migraine in Latvia and Lithuania. On the other hand, migraineurs should be surveyed for refining the estimates on health care utilization, the number of days missed from work and the number of days lost due to impairment. Conducting such surveys are not only time-consuming and costly, but in addition to careful design and implementation they also require large sample sizes. To increase the reliably and validity of survey-based estimates, the sample size should preferably be larger than what is currently available in the literature for European countries. At the same time, their methodology should be carefully designed, for example, any prevalence rate estimate shall be derived from population-based samples, it requires a validated questionnaire and trained interviewers to conduct telephone or face to face structured interviews.

## Conclusions

In this study we delivered a conservative estimate for the economic cost of migraine in Latvia and Lithuania; the prevalence rate of migraine and the number of lost workdays adopted from the literature can be considered as lower bounds for those items. We found that mean per-person total cost of migraine is €801 annually in Latvia, and €721 in Lithuania. Mean per-person direct cost is €206 in Latvia and €178 in Lithuania. In both countries less than 30% of total cost is direct cost; cost related to a wide array of medical services and interventions. Mean per-person indirect cost for unemployed migraineurs is €11,111 in Latvia, and €10,085 in Lithuania; this cost is related to reduced workforce participation. Mean per-person indirect cost for employed migraineurs is €564 in Latvia, and €521 in Lithuania, this cost is related to absenteeism and impairment while at work. The total cost of migraine is €112.26 million in Latvia, corresponding to 0.42% of Latvia’s GDP. The total cost of migraine is €149.62 million in Lithuania, corresponding to 0.35% of Lithuania’s GDP. Around 70% of the total cost is indirect cost, the huge majority of which is related to lost work either due to absence or impairment. Our findings unambiguously reveal that the financial burden of migraine imposed on the society as a whole is substantial in Latvia and Lithuania.

In the absence of cost-benefit analysis it is too early to formulate relevant policy recommendations. As for speculation, there might be opportunities for cost-effective interventions. Patients treated with medications and procedures which alleviate the symptoms of migraine will have higher quality of life, increased work productivity and reduced impairment in all aspects of life. Although improvements in care for patients with migraine, such as wider availability of various procedures or innovative medications will significantly increase direct costs, this cost increase might be outweighed by lower migraine-related productivity loss. Given that the prevalence of migraine is the highest in the most productive years of life, preventive treatment and effective headache management might deliver significant benefits on both personal and professional level, not only to the ones affected by the migraine but to the entire society and national economy. With this study we aimed at raising awareness of the considerable financial burden of migraine and the high unmet need of migraineurs in two Baltic countries. Further cost-benefit analysis could illuminate interventions that lower the financial burden of migraine on the society and economy as a whole.

## Additional files

Additional file 1: Deriving the annual number of days missed from work. This file provides detailed information on translating the various absenteeism estimates [6, 7, 14, 27, 32, 35, 37] into annual figures. (DOCX 14 kb)

Additional file 2: Deriving the annual number of days lost due to impairment. This file provides detailed information on translating the various presenteeism estimates [6, 7, 14, 27] into annual figures. (DOCX 14 kb)

Additional file 3: Notes to unit cost price estimations. This file provides detailed information on estimating unit cost prices in Latvia and Lithuania [64]. (DOCX 17 kb)

Additional file 4: Annual medication cost of selected acute migraine therapies in Latvia (LV) and Lithuania (LT). This file provides detailed information on estimating the annual medication cost of selected migraine therapies with paracetamol, ibuprofen, aspirin, and triptan. The file lists the active substance, the price per package, the defined daily dose, the maximum dosage, and the assumptions on the number of monthly headache days and medication usage. (DOCX 18 kb)

## Data Availability

The data used during this study are available from public sources identified in the paper. All data analysed during this study are included in this published article and its supplementary information files; the web links to the raw data on which the analysis is based are included in the reference list, see references [43–45, 48–54, 59, 62, 64–75].

## Abbreviations

AMPP study: American Migraine Prevalence and Prevention study
CM: Chronic migraine
CT: Computed tomography
DRG: Diagnostic Related Grouping
ECG: Electrocardiogram
EEG: Electroencephalogram
EM: Episodic migraine
GDP: Gross Domestic Product
IBMS-I: First International Burden of Migraine Study
IBMS-II: Second International Burden of Migraine Study
ICHD: International Classification of Headache Disorders
LT: Lithuania
LV: Latvia
MHDs: Monthly headache days
MRI: Magnetic resonance imaging
NSAIDs: Nonsteroidal Anti-inflammatory Drugs

## Footnotes

1 The search term for identifying systematic reviews on migraine prevalence was as follows. The abstract, title or key word should include the following terms: 1) migraine; 2) prevalence; 3) “systematic review”; or “systematic literature”; or “all existing evidence”. The search was performed on 17 January 2019; only journal articles published in English were retrieved.

2 Studies were excluded if they used a prevalence estimate from a previous systematic review (*n* = 3); reviewed studies from a specific region other than Europe (*n* = 2); focused on patients with chronic migraine only (*n* = 3); discussed the comorbidities of migraine (*n* = 15), had other headache, pain or disorder in their focus (*n* = 5); reviewed aspects of the treatment method (*n* = 4); or had a completely different focus (*n* = 10).

3 Search term for identifying studies on migraine prevalence is Latvia and Lithuania was as follows. The abstract, title or key word should include the following terms: 1) migraine; 2) prevalence; 3) Latvia or Lithuania or Estonia. The search was performed on 22 January 2019; only journal articles published in English were retrieved.

4 Several studies use data from these surveys for purposes other than estimating migraine-related health care resource utilization (e.g., [26]); these studies are excluded from Table 2.

5 The estimate of Vo et al. [27] is derived from the data reported in Table 2 in [27]. This estimate includes the number of general/family practitioner visits, neurologist visits psychiatrist visits, ED visits and hospitalizations, and excludes any other health care provider visits. The estimate of Bloudek et al. [28] is derived from the data reported in Table 3 in [28]; the proportion of participants reporting one or more visits was multiplied the mean number of events of those reporting one or more visits. This estimate includes the number of primary care physician visits, neurologist/headache specialist visits, nurse practitioner/physician assistant visits, other specialist visits, emergency room visits and hospitalizations.

6 The search term defined was as follows: the title and/or the abstract should include migraine, medication and cost, while Latvia or Lithuania might be part of any field, including the text. This search resulted in only one study for Lithuania [20]. As a result, we have also performed an extended search where the title and/or the abstract included migraine, medication and cost without any geographic limitations. The title of the 126 articles identified in this way has been screened; this extended search yielded no additional studies. The search was performed on 5 March 2019; only journal articles published in English were retrieved and screened.

7 Number of patients with migraine x proportion of patients with chronic migraine x annual cost of therapy with the cheapest triptan = 207.044 x 10.55% x €10.83 = €278,018.

8 In both countries the number of episodic migraineurs being unemployed were estimated as follows: number of all migraineurs x the proportion of episodic migraineurs x unemployment rate × 1.028, where the proportion of episodic migraineurs was assumed to be 95.12% as reported in Table 3, and the multiplier of 1.028 reflects the higher likelihood of being unemployed if an individual suffers from episodic migraine as reported in [14]. From these unemployed migraineurs only the increment was considered as being unemployed due to their condition (number of all migraineurs x the proportion of episodic migraineurs x unemployment rate × 0.028). The number of chronic migraineurs being unemployed were estimated as follows: number of all migraineurs x the proportion of chronic migraineurs x unemployment rate × 1.19, where the proportion of chronic migraineurs was assumed to be 4.88% as reported in Table 3, and the multiplier of 1.19 reflects the higher likelihood of being unemployed if an individual suffers from chronic migraine as reported in [14]. From these unemployed migraineurs only the increment was considered as being unemployed due to their condition (number of all migraineurs x the proportion of chronic migraineurs x unemployment rate × 0.19). For the remaining migraineurs the primary reason of their unemployment are conditions other than migraine.
