# Supplementary material for: Economic burden of migraine in Latvia and Lithuania: direct and indirect costs
Source: BMC Public Health. 2019 Sep 9;19:1242. doi: 10.1186/s12889-019-7461-2 (PMC6734255; doi:10.1186/s12889-019-7461-2)
Supplement: Supplementary file 1 — Deriving the annual number of days missed from work. This file provides detailed information on translating the various absenteeism estimates [6, 7, 14, 27, 32, 35, 37] into annual figures. (DOCX 14 kb) [file 12889_2019_7461_MOESM1_ESM.docx]

## Additional file 1.

## Deriving the annual number of days missed from work

| **Study** | **Calculation** |
| --- | --- |
| Munakata et al. [6]^a^ | Munakata et al. [6] report 13.7 hours lost per year due to absenteeism, for patients with migraine. This figure translates into 13.7/8= 1.71 workdays lost per year. |
| Stewart et al. [14] ^b^ | Stewart et al. [14] report hours per worker per week missed from work. These estimates were multiplied by 52 (number of weeks per year) and divided by 8 (number of working hours per day) to arrive at annual estimate. Patients were considered to suffer from episodic migraine if they had low (<10 days of headache in 3 months), moderate (10-29 days of headache in 3 month) or high frequency headaches (30-44 days of headache in 3 months). Patients were considered to suffer from chronic migraine if they had more than 45 days of headache in 3 months. For episodic migraine weighted average of the three patient groups is calculated: $(0.3x\frac{52}{8}x3,697+0.6x\frac{52}{8}x1,949+1x\frac{52}{8}x283$) / (3,697+1,949+283) =2.81 days. Chronic migraineurs lost $0.8 x\frac{52}{8}=5.20 work$days. For all migraineurs weighted average of the four patient groups is calculated: $0.3x\frac{52}{8}x 3,697+0.6x\frac{52}{8}x1,949+1 x\frac{52}{8}x 283+0.8x\frac{52}{8}x275$) / (3,697+1,949+283+275) =2.91 days. |
| Steiner et al. [12]^c^ | Steiner et al. [12] report the days lost in preceding 3 months which has been multiplied by four to arrive at an annual estimate. This figure includes both the workdays lost completely and the workdays with productivity reduced to 50% or more of the expected productivity. Figures for patient subgroups with episodic and chronic migraine is not reported. |
| Ayzenberg et al. [7]^d^ | Ayzenberg et al. [7] report the days lost in preceding 3 months which has been multiplied by four to arrive at the annual days per year figures. Figures for patient subgroups with episodic and chronic migraine is not reported; in their sample participants with headache on 15 days or more include both patients with migraine and tension-type headache. |
| Vo et al. [29]^e^ | Vo et al. [29] report the percentage of time missed which has been multiplied by 252 working days to arrive at the annual days per year figures [55]. The figures shown in the table reflect the differences between patients with migraine and the control group (patients without migraine). For episodic migraine weighted average of two patient groups (patients with 4-7 and 8-14 monthly headache days) is calculated: [(7.98*x*66 + 22.19*x*32)/(66+32) – 9.45]/100 x 252 = 7.99 days. Chronic migraineurs missed (19.65-9.45)/100 *x* 252 = 25.70 days from work. All migraineurs missed (14.43-9.45)/100 *x* 252 = 12.55 days from work. |
| Vo et al. [44]^f^ | Vo et al. [44] find that migraineurs missed 2.3 working days on average, which translates into 12*x*2.3=27.6 days per year. The respective figure is 12*x*4.4=52.8 days for patients with chronic migraine. For episodic migraine weighted average of two patient groups (patients with 4-7 and 8-14 monthly headache days) is calculated: (1.2*x*1,500 + 2.1 x 1,500)/(1,500+1,500) = 1.65 days per month, and thus 12*x*1.65=19.8 days per year. |
| Martelletti et al. [33]^g^ | Martelletti et al. [33] document that respondents in employment had an average 2.4 paid sick days which translates into 12*x*2.4=28.8 paid sick days per year. |
