# Supplementary material for: Economic burden of migraine in Latvia and Lithuania: direct and indirect costs
Source: BMC Public Health. 2019 Sep 9;19:1242. doi: 10.1186/s12889-019-7461-2 (PMC6734255; doi:10.1186/s12889-019-7461-2)
Supplement: Supplementary file 2 — Deriving the annual number of days lost due to impairment. This file provides detailed information on translating the various presenteeism estimates [6, 7, 14, 27] into annual figures. (DOCX 14 kb) [file 12889_2019_7461_MOESM2_ESM.docx]

## Additional file 2.

## Deriving the annual number of days lost due to impairment

| **Study** | **Calculation** |
| --- | --- |
| Munakata et al. [6] ^a^ | Munakata et al. [6] report 48.3 hours lost per year due to presenteeism, for patients with migraine. This figure translates into 48.3/8= 6.04 days lost per year. |
| Stewart et al. [14] ^b^ | Stewart et al. [14] report hour-equivalent of headache-related reduced performance per worker per week. These estimates were multiplied by 52 (number of weeks per year) and divided by 8 (number of working hours per day) to arrive at an annual estimate. Patients were considered to suffer from episodic migraine if they had low (<10 days of headache in 3 months), moderate (10-29 days of headache in 3 month) or high frequency headaches (30-44 days of headache in 3 months). Patients were considered to suffer from chronic migraine if they had more than 45 days of headache in 3 months. For episodic migraine weighted average of the three patient groups is calculated: $(0.8x\frac{52}{8}x3,697+1.9x\frac{52}{8}x1,949+2.8x\frac{52}{8}x283$) / (3,697+1,949+283) =8.17 days. Chronic migraineurs lost $3.8 x\frac{52}{8}=24.70$days due to impairment. For all migraineurs weighted average of the four patient groups is calculated: $0.8x\frac{52}{8}x 3,697+1.9x\frac{52}{8}x1,949+2.8 x\frac{52}{8}x 283+3.8x\frac{52}{8}x275$) / (3,697+1,949+283+275) =8.90 days. |
| Ayzenberg et al. [7]^c^ | Ayzenberg et al. [7] report the days lost in preceding 3 month which has been multiplied by four to arrive at an annual estimate. Figures for patient subgroups with episodic and chronic migraine is not reported; in their sample participants with headache on 15 days or more include both patients with migraine and tension-type headache. |
| Vo et al. [29]^d^ | Vo et al. [29] report the percentage of impairment while at work for three patient groups: patients with 4-7 headache days per month, patients with 8-14 headache days per month, and patients with more than 15 days of headache per month (chronic migraine). To translate the percentage of impairment while at work into days missed from work on an annual basis, we perform the following transformations. First, the number of working days with migraine per year is calculated in each patient group. For each group, we assume that patients have the minimum number of days with headache per month, 4, 8, 15 days, respectively, which translates into 48, 96 and 180 number of days with headache per year. Second, we split the headache days between those occurring on working days, and those occurring on other days (weekends, holidays, sick leave). The maximum number of working days per year is assumed to be 252 [55]. The number of holidays is considered as 20 days. The number of days with sick leave is derived from the absenteeism estimates in [29]; the authors report the percentage of work time missed for the three patient groups, 7.98%, 22.19% and 19.65%, respectively. The number of working days is derived as follows: (maximum number of working days – holidays) *x* (1-percentage of work time missed). Third, the number of headache days while at work is calculated for each patient group: number of headache days *x (*number of working days/365. For patients with 4-7 headache days per month the estimation is as follows: 48*x*((252-20)*x*(1-0.0798))/365=28.07 working days with migraine per year. For patients with 8-14 headache days per month the estimation is as follows: 96*x*((252-20)*x*(1-0.2219))/365=47.48 working days with migraine per year. For patients with more than 15 days of headache per months the estimation is as follows: 180*x*((252-20)*x*(1-0.1965))/365= 91.93 working days with migraine per year. Finally, we multiply the number of working days with migraine with the incremental impairment, the difference in the impairment between patients with migraine and the control group (patients without migraine). Patients with 4-7 headache days per month miss 28.07*x(*29.38%-20.97%)=2.36 days from work each year due to impairment. Patients with 8-14 headache days per month miss 47.48*x(*33.79%-20.97%)=6.09 days from work each year due to impairment. Patients with chronic migraine miss 91.93*x(*49.38%-20.97%)=26.12 days from work each year due to impairment. In Table 6, for episodic migraine weighted average of two patient groups (patients with 4-7 and 8-14 monthly headache days) is calculated: [(2.36 *x* 64 + 6.09 *x* 29)/(64+29)= 3.52 days. Entry for all migraine is calculated as the weighted average of the three patient groups: [(2.36 *x* 64 + 6.09 *x* 29 + 26.12 *x* 32)/(64+29+32)= 9.31 days. |
