# Supplementary material for: Economic burden of migraine in Latvia and Lithuania: direct and indirect costs
Source: BMC Public Health. 2019 Sep 9;19:1242. doi: 10.1186/s12889-019-7461-2 (PMC6734255; doi:10.1186/s12889-019-7461-2)
Supplement: Supplementary file 3 — Notes to unit cost price estimations. This file provides detailed information on estimating unit cost prices in Latvia and Lithuania [64]. (DOCX 17 kb) [file 12889_2019_7461_MOESM3_ESM.docx]

## Additional file 3.

## Notes to unit cost price estimations

| ^a^ | In Latvia, general practitioners receive €19.2 fixed annual per capita fee (€1.6x12). At each visit there is a patient co-payment of €1.42. The unit cost for one visit was derived as the ratio of the annual per capita fee and the number of contacts in primary care per one population (5.9) [64], plus the patient co-payment. Privately funded primary care physician fees vary greatly, they range from €15 to €65.40. In sensitivity test, we use the fee of the first visit (€28.46) at one large Latvian health care provider [65]. |
| --- | --- |
| ^b^ | In Lithuania, general practitioners receive fixed annual per capita fee (€23.19 for persons aged 18-34 years; €29.05 for persons aged 35-49 years; €45.41 for persons aged 50-65 years). As mean age of patients with migraine in Lithuania is 42.5 [18], the unit cost for one visit was derived as the ratio of the annual fee per persons aged 35-49 and the number of visits in primary care per one population (6.47) [66]. |
| ^c^ | The fee of publicly funded visit is €14.04 with a patient co-payment of €4.27. The fee of privately funded first visit ranges from €40-50; the fee for repetitive visit is €35. |
| ^d^ | Fee of consulting a neurologist in tertiary care (university hospital) institution. Fee for consulting a neurologist in secondary care institution is €15.26; fee for the first consultation with private neurologist is €49; fee for repetitive consultation with private neurologist is €31. |
| ^e^ | The fee of publicly funded visit is €9.65 with a patient co-payment of €4.27. The fee of privately funded visit ranges from €30-45. |
| ^f^ | The fee of publicly funded visit in secondary level. The fee of publicly funded visit is €26.11 in tertiary level. The fee is €36.55 if additional services provided, e.g. biopsy. |
| ^g^ | The fee of publicly funded visit is €10.04 with a patient co-payment of €4.27. The fee of privately funded first visit ranges from €40-62.2. |
| ^h^ | There is no separate fee for visiting a pain specialist, the fee is the same as for consulting a neurologist |
| ^i^ | Fee of privately funded visit. Public funding is only available for children psychologist. |
| ^j^ | The fee of publicly funded visit is €3.42 per case (primary level only). |
| ^k^ | The fee of publicly funded visit is €4.34 with a patient co-payment of €4.27. The fee of privately funded visit ranges from €35-45. |
| ^l^ | The fee of publicly funded visit in case of primary assessment in secondary level. The fee of publicly funded visit is € 35.55 in case of primary assessment in tertiary level. |
| ^m^ | Costs of emergency visits vary greatly across hospitals in Latvia [67]. At the lower end we find the high-volume state hospitals (e.g., Rīgas 2. Slimnīca, Paula Stradiņa Klīniskā Universitātes Slimnīca, Rīgas Austrumu Klīniskās Universitātes Slimnīca); visit costs vary from €36.28- to €39.89. At the higher end we find the remote and small hospitals (e.g., Krāslavas Slimnīca, Preiļu Slimnīca); mean visit costs might be as high as €275.55-406.72. As a conservative estimate we use the unit cost prices of €38.79 at Paula Stradiņa Klīniskā Universitātes Slimnīca. In sensitivity test we use the unit cost prices of €117.57, the average across general hospitals. |
| ^n^ | As the Lithuanian health care system uses Diagnostic Related Grouping (DRG), emergency room visits are included in the hospitalization case prices. For cases where the patients are dismissed after visiting the emergency room, fee of consulting a neurologist in tertiary care (university hospital) institution is used. The fee of consulting a neurologist in a secondary care institution is lower, €15.26. |
| ^o^ | Fee per day in regional, publicly funded hospital, of which €10 is patient co-payment. Fee per day in university hospital is €102.01, while in local hospital is €73.03. |
| ^p^ | Price per treatment case when status is not complicated (DRG code B77B), assuming 2-12 days of hospitalizations. Price for an additional day of hospitalization when status is not complicated is €31.93 per day. The price per treatment case when status is complicated (DRG code B77A) is €447.08, assuming 2-16 days of hospitalizations. Price for an additional day of hospitalization when status is complicated is €68.98 per day. |
| ^q^ | Per day. |
| ^r^ | MRI without contrast agent. The public funding is €84.36 with a patient co-payment of €28.46. The fee of privately funded MRI is €115 [68]. In public setting, the fee of MRI with contrast agent is €104.46 with a patient co-payment of €35.57. |
| ^s^ | CT without contrast agent. The public funding is €11.42 with a patient co-payment of €14.23. The fee of privately funded CT without contrast agent in outpatient care is €93 (1. Slimnica, 2019). |
| ^t^ | The public funding is €21.17 with a patient co-payment of €4.27. The fee of privately funded EEG in outpatient care is €42 [68]. |
| ^u^ | Included in the fee of visiting a neurologist, no additional funding is provided. When electroencephalography is performed, the fee is €21.68 Eur in secondary care institution and €35.35 in tertiary care (university hospital). The former figure is used in this study. |
| ^v^ | Without ECG description. The lower bound of public funding is €1.87 with a patient co-payment of €4.27. The fee of privately funded ECG in outpatient care is €12 [68]. |
| ^w^ | Included in the fee of visiting a cardiologist, no additional funding is provided. As a proxy we use the fee of visiting a cardiologist in secondary care institution. The fee of visiting a cardiologist in tertiary care (university hospital) institution would be €26.11. |
| ^x^ | The public funding for two projections is €10.65 with a patient co-payment of €2.85. The fee of privately funded neck X-ray in outpatient care is €14.85 [68]. |
| ^y^ | Included in the fee of visiting a radiologist, no additional funding is provided. As a proxy we use the fee of visiting a radiologist in secondary care institution. The fee of visiting a radiologist in tertiary care (university hospital) institution would be €26.11. |
| ^z^ | General blood test including leucocyte count. For migraine patients other typical blood test examinations include, generally, to rule other causes of migraine symptoms: iron level €0.90; C-reactive protein (CRP) test €1.80; anti streptolizin test €1.95; thyroid-stimulating hormone test €4.25 [69]. |
| ^α^ | Average fee for blood test in the private sector. In Lithuania, blood test is typically included in the general practitioner’s fee, no additional funding is provided. |
| ^β^ | Paid out of pocket. |
| ^γ^ | Information about prices was retrieved from Premium Medical [70]. |
| ^δ^ | Within a range of €20-35. |
| ^ε^ | Information about prices was retrieved from Vivendi [71]. |
| ^ζ^ | In Lithuania, there is no separate cost for this procedure; it is included in the price of the botulinum toxin A injections. The procedure is performed quarterly and is paid out of pocket. |
| ^η^ | (Proportion of episodic migraineurs *x* the cost of the cheapest annual therapy with over the counter medications) + (Proportion of chronic migraineurs *x* the cost of the cheapest annual therapy with triptans) = (89.45% x €10.21) + (10.55% x €29.67) = €12.26. For annual cost of therapies with various over-the-counter and prescription medications and related assumptions see Additional file 2. |
| ^θ^ | Mean per patient medication cost estimate used in the sensitivity test. (Proportion of analgesics users *x* the cost of the cheapest annual therapy with analgesics) + (Proportion of NSAID users *x* the cost of the cheapest annual therapy with NSAIDs) + (Proportion of triptan users *x* the cost of the cheapest annual therapy with triptans) = (67.63% x €16.90) + (48.02% x €10.21) + (16.75% x €29.67) = €21.31. Further details are reported when performing the sensitivity tests. |
| ^ι^ | (Proportion of episodic migraineurs *x* the cost of the cheapest annual therapy with over the counter medications) + (Proportion of chronic migraineurs *x* the cost of the cheapest annual therapy with triptans) = (89.45% x €9.21) + (10.55% x €10.83) = €9.39. For annual cost of therapies with various over-the-counter and prescription medications and related assumptions see Additional file 2. |
