# Supplementary material for: Economic burden of migraine in Latvia and Lithuania: direct and indirect costs
Source: BMC Public Health. 2019 Sep 9;19:1242. doi: 10.1186/s12889-019-7461-2 (PMC6734255; doi:10.1186/s12889-019-7461-2)
Supplement: Supplementary file 4 — Annual medication cost of selected acute migraine therapies in Latvia (LV) and Lithuania (LT). This file provides detailed information on estimating the annual medication cost of selected migraine therapies with paracetamol, ibuprofen, aspirin, and triptan. The file lists the active substance, the price per package, the defined daily dose, the maximum dosage, and the assumptions on the number of monthly headache days and medication usage. (DOCX 18 kb) [file 12889_2019_7461_MOESM4_ESM.docx]

## Additional file 4.

## Annual medication cost of selected acute migraine therapies in Latvia (LV) and Lithuania (LT)

|  | **Active substance** | **Price per package LV vs. LT** | **DDD/**  **Max** | **Daily dosage (mg)** | **Annual cost**  **LV vs LT** |
| --- | --- | --- | --- | --- | --- |
| **Paracetamol (acetaminophen)** | | | | | |
| Paramax Rapid 500 mg, Vitabalans Oy, Finland, 30 tablets | Paracetamol | 1.76 (LV) | DDD | 3000 | 16.90 (LV) |
| Paramax Rapid 500 mg tablets, Vitabalans Oy, Finland, 30 tablets | Paracetamol | 1.76 (LV) | Max | 4000 | 22.53 (LV) |
| Paracetamol Accord 500mg tablets, Accord Healthcare, 20 tablets | Paracetamol | 0.84 (LT) | DDD | 3000 | 12.10 (LT) |
| Paracetamol Accord 500mg tablets, Accord Healthcare, 20 tablets | Paracetamol | 0.84 (LT) | Max | 4000 | 16.13 (LT) |
| Paracetamol-Grindeks 500 mg tablets, 20 tablets | Paracetamol | 2.04 (LV)  2.04 (LT) | DDD | 3000 | 29.38 (LV) 29.38 (LT) |
| Paracetamol-Grindeks 500 mg tablets, 20 tablets | Paracetamol | 2.04 (LV)  2.04 (LT) | Max | 4000 | 39.17 (LV)  39.17 (LT) |
| **Ibuprofen** | | | | | |
| Ibumax 400 mg tablets, Vitabalans Oy, Finland, 100 tablets | Ibuprofen | 7.09 (LV)  6.40 (LT) | DDD | 1200 | 10.21 (LV)  9.22 (LT) |
| Ibumax 400 mg tablets, Vitabalans Oy, Finland, 100 tablets | Ibuprofen | 7.09 (LV)  6.40 (LT) | Max | 3400 | 28.93 (LV)  26.11 (LT) |
| **Aspirin (acetylsalicylic acid)** | | | | | |
| Aspirin 500 mg tablets, UAB Bayer, Lithuania, 100 tablets | Acetylsali-cylic acid | 11.84 (LV)  11.39 (LT) | DDD | 3000 | 34.10 (LV)  32.80 (LT) |
| Aspirin 500 mg tablets, UAB Bayer, Lithuania, 100 tablets | Acetylsali-cylic acid | 11.84 (LV)  11.39 (LT) | Max | 4000 | 45.47 (LV)  43.74 (LT) |
| **Triptans** |  |  |  |  |  |
| Almotriptan 12.5 mg tablets, Zentiva, 3 tablets | Almotriptan malate | 15.00 (LV)  11.87(LT) | DDD/Max | 12.5 | 60 (LV)  47.48 (LT) |
| Cinie 100 mg tablets, Zentiva, 6 tablets | Sumatriptan | 29.67 (LV) | DDD | 50 | 29.67 (LV) |
| Cinie 100 mg tablets, Zentiva, 6 tablets | Sumatriptan | 29.67 (LV) | Max | 200 | 118.68 (LV) |
| Sumatriptan 100mg tablets, Stada, 6 tablets | Sumatriptan | 10.38 (LT) | DDD | 50 | 10.83 (LT) |
| Sumatriptan 100mg tablets, Stada, 6 tablets | Sumatriptan | 10.38 (LT) | Max | 200 | 43.32 (LT) |
| Migard 2.5 mg tablets, Menarini, 2 tablets | Frovatriptan | 11.87 (LV)  8.47 (LT) | DDD | 2.5 | 71.22 (LV)  50.82 (LT) |
| Migard 2.5 mg tablets, Menarini, 2 tablets | Frovatriptan | 11.87 (LV)  8.47 (LT) | Max | 5 | 142.44 (LV)  101.64 (LT) |
| Relpax 40mg tablets, Pfizer, 4 tablets | Eletriptan | 25.11 (LV) | DDD | 40 | 75.33 (LV) |
| Relpax 40mg tablets, Pfizer, 4 tablets | Eletriptan | 25.11 (LV) | Max | 80 | 150.66 (LV) |
| Zolmitriptan 2.5mg tablets, Glenmark, 6 tablets | Zolmitriptan | 35.49 (LT) | DDD | 2.5 | 70.89 (LT) |
| Zolmitriptan 2.5mg tablets, Glenmark, 6 tablets | Zolmitriptan | 35.49 (LT) | Max | 10 | 283.92 (LT) |

LV=Latvia, LT=Lithuania, DDD=Defined daily dosage

Becker [36] concludes that current acute migraine medications can be organized into four treatment strategies. Strategy 1 covers patients with attacks of mild to moderate severity; paracetamol (acetaminophen) and/or nonsteroidal anti-inflammatory drugs (NSAIDs) are suggested for these patients. Strategy 2 covers patients with severe attacks and patients who do not respond to strategy 1; these patients shall take triptans. Strategy 3 shall be applied for patients where their migraine attacks are refractory to strategies 1 and 2. Strategy 4 is to be applied for patients with contraindications to vasoconstricting drugs. In the table, annual cost of several therapies is shown for patients treated with paracetamol, NSAIDs and triptans (Strategy 1 and 2). The following assumptions were made:

1. Patients suffer from 4 monthly headache days which translates into 48 annual headache days.
2. Paracetamol, ibuprofen and aspirin are taken each day with headache. Triptans are taken once per attack, assuming monthly one attack.
3. Patients select the cheapest available medication. Information about medications and prices were retrieved from the Medicinal Product Register of Latvia for Latvia [72] and from vaistail.lv for Lithuania [73].
4. Patients consider both dosage and number of tablets per package when searching for the cheapest medication with the desired active substance.
5. Patients take either the defined daily dose (DDD) or the maximum dosage per day, as indicated in the table. DDD data were retrieved from WHO [74], maximum dosage is based on Latvijas Ārsts [75].
